# A Gut‐Brain Axis Underlying Succinate‐Associated Compulsive‐Like Behavior

**DOI:** 10.1002/advs.77836

**Published:** 2026-09-27

**Authors:** Yi Li, Ying‐Dan Zhang, Wei Wang, Xin‐Yi Wu, Di Li, Xin Gong, Fei Tian, Meng‐Yu Dai, Jian Gao, Zi‐Feng Zheng, Zi‐Yang Bi, Dong‐Dong Shi, Zhen Wang

**Affiliations:** ^1^ Shanghai Mental Health Center Shanghai Jiao Tong University School of Medicine Shanghai China; ^2^ School of Psychology Shanghai Jiao Tong University Shanghai China; ^3^ School of Integrative Medicine Shanghai University of Traditional Chinese Medicine Shanghai China; ^4^ Shanghai Key Laboratory of Mental Disorders Translational Research Shanghai Mental Health Center, Shanghai Jiao Tong University School of Medicine Shanghai China

**Keywords:** gut‐brain axis, microglia, obsessive‐compulsive disorder, succinate, SUCNR1, vagus nerve

## Abstract

Emerging evidence indicates that gut microbiota influences host physiology and behavior through the microbiota‐gut‐brain axis, potentially contributing to the pathogenesis of obsessive‐compulsive disorder (OCD). Our preliminary research demonstrated that mice colonized with fecal microbiota from OCD patients developed compulsive‐like behaviors, associated with a significant increase in the microbial metabolite succinate. However, the mechanisms linking succinate to compulsive‐like behavior remain to be fully elucidated. Here, we demonstrate that succinate is associated with significant compulsive‐like behaviors. Succinate is associated with disruptions of glutamatergic neurons within the dorsal medial prefrontal cortex (dmPFC), and we characterize dual pathways linked to the behavioral abnormality: both by dmPFC microglial engagement via the succinate cognate receptor SUCNR1, and by gut‐to brain signaling conveyed through vagal afferents and the 5‐HT_4_ receptor. Pharmacological modulation of the succinate metabolism pathway through succinate dehydrogenase inhibition ameliorates compulsive‐like behavior in *Hoxb8* ^−/−^ OCD‐model mice. Circulating succinate levels significantly distinguish OCD patients from healthy controls with an AUC of 0.73, and correlate with prefrontal cortex activity on functional imaging. Collectively, our study delineates a gut‐brain axis implicating succinate in central nervous system dysregulation associated compulsive‐like behavior, elucidates novel immunometabolism and neural circuit mechanisms underlying OCD pathogenesis, and identifies druggable targets for potential precise interventions.

## Graphical abstract

Gut‐derived succinate is associated with an excitatory‐inhibitory imbalance in dmPFC glutamatergic neurons, involving microglial SUCNR1 activation and vagal afferent signaling via upregulated gut 5‐HT_4_ receptors along the DVC‐CeA‐dmPFC circuit, changes that are linked to excessive compulsive‐like behavior. Serum succinate emerges as a candidate biomarker for obsessive‐compulsive disorder, and targeting succinate metabolism may offer therapeutic potential.

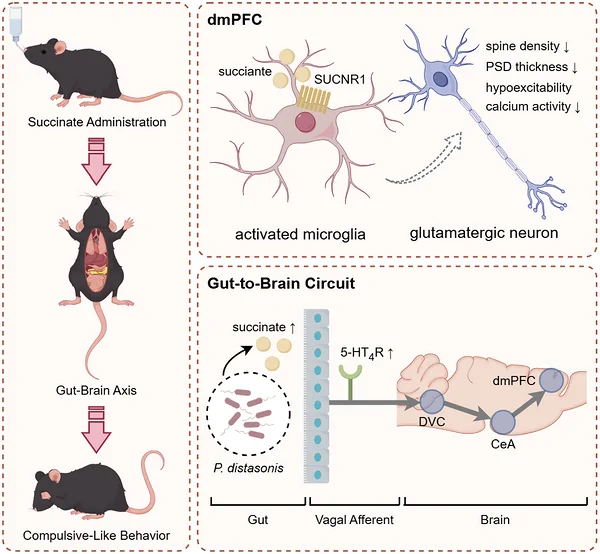

## 1 Introduction

Obsessive‐compulsive disorder (OCD) is a chronic psychiatric condition characterized by intrusive thoughts and repetitive behaviors [1], affecting approximately 3% of the global population [2]. Despite advances in pharmacological and behavioral therapies, a substantial proportion of patients remain treatment‐resistant, highlighting the need for novel therapeutic targets [3]. In recent years, the microbiota‐gut‐brain axis has emerged as a critical interface linking gut microbial communities to central nervous system (CNS) function and behavior [4]. Accumulating evidence suggests that gut microbiota dysbiosis may contribute to the pathophysiology of various neuropsychiatric disorders [5], and represents a promising therapeutic potential [6].

Several clinical studies have identified distinct gut microbiota profiles in patients with OCD compared to healthy controls [7, 8]. Reduced gut microbiome α‐diversity have been consistently reported in both adult [9] and pediatric [10] OCD cohorts. Furthermore, a two‐sample Mendelian randomization analysis provides evidence supporting a potential causal role of specific gut microbiota in OCD [11]. Critically, our prior preclinical investigations [12] demonstrated that fecal microbiota transplantation (FMT) from drug‐naïve OCD patients into mice recapitulated pronounced compulsive‐like behavioral phenotypes, providing causal evidence implicating gut dysbiosis in OCD pathogenesis. Subsequent metabolomic profiling of FMT models pinpointed succinate, a key intermediate linked to the Krebs cycle [13], as a significantly elevated metabolite both in circulation and CNS with putative pathogenic relevance to OCD pathophysiology. While succinate has been implicated in mitochondrial function [14] and immune response [15], the precise mechanisms by which gut‐derived succinate is linked to CNS dysfunction and aberrant compulsive‐like behavioral manifestations through the gut‐brain axis remain incompletely understood.

Given that gut‐derived metabolites can modulate CNS activity through both direct (via crossing the blood‐brain barrier) and indirect pathways (by activating gut‐innervating peripheral afferent nerves, particularly vagal afferents, which relay signals to the CNS) [16, 17], the present study investigates the association of succinate with both central neuropathology and peripheral gut‐brain neural signaling in the context of succinate‐associated compulsive‐like behaviors. Building upon these findings, this work aims to elucidate the complex interplay between gut‐derived pathogenic metabolites and CNS dysfunction, unraveling the molecular and neural circuit mechanisms underlying succinate's contribution to OCD‐relevant phenotypes. Our findings are anticipated to provide crucial mechanistic insights, informing the development of novel, mechanism‐based therapeutic strategies for OCD management.

## 2 Results

### 2.1 Gut‐Derived Succinate is Associated With Compulsive‐Like Behaviors in Mice

**FIGURE 1:**
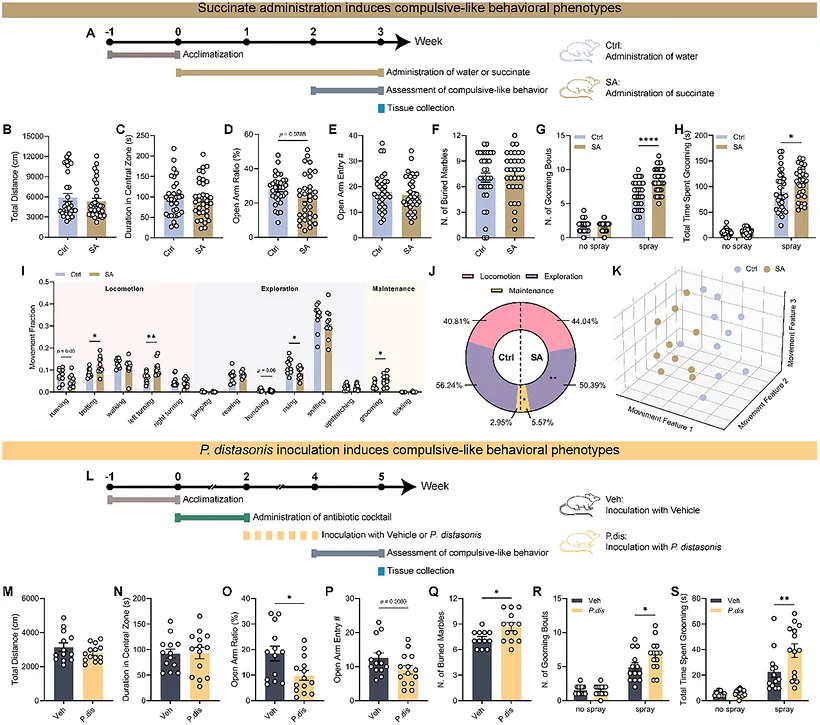
Succinate administration and *P. distasonis* inoculation are associated with compulsive‐like behaviors. (A) Experimental design for assessing the effects of succinate administration on behavioral phenotypes. (B–E) Locomotor activity and anxiety‐like behaviors assessed by total distance traveled in the open field (B), time spent in the central zone (C), proportion of time spent in the open arms (D), and frequency of entries into the open arms (E). (*n* = 32 Ctrl, *n* = 33 SA). (F–H) Compulsive‐like behaviors assessed by number of buried marbles (F), number of grooming bouts (G), and total grooming duration (H). (*n* = 32 Ctrl, *n* = 33 SA). (I and J) Movement fraction (I) and cluster proportion (J) of spontaneous behavior. (*n* = 10 Ctrl, *n* = 10 SA). (K) PCA of movement features during spontaneous behavior. (*n* = 10 Ctrl, *n* = 10 SA). (L) Experimental design for assessing the effects of *P. distasonis* inoculation on behavioral phenotypes. (M–P) Locomotor activity and anxiety‐like behaviors assessed by total distance traveled in the open field (M), time spent in the central zone (N), proportion of time spent in the open arms (O), and frequency of entries into the open arms (P). (*n* = 13 Veh, *n* = 14 P.dis). (Q–S) Compulsive‐like behaviors assessed by number of buried marbles (Q), number of grooming bouts (R), and total grooming duration (S). (*n* = 13 Veh, *n* = 14 P.dis). Each dot represents an individual mouse. Data are represented as mean ± SEM. Statistical significance was determined by unpaired two‐tailed *t*‐test (C, D, E, I, M, N, O, Q), Mann‐Whitney test (B, F, P), or two‐way ANOVA (G, H, J, R, S). *p < 0.05; **p < 0.01; ***p < 0.001; ****p < 0.0001.

To investigate whether gut‐derived succinate is associated with compulsive‐like behaviors, mice received ad libitum sodium succinate in sterile drinking water and underwent behavioral tests (Figure 1A). Succinate‐administered (SA) mice displayed significantly increased grooming bouts (Figure 1G) and prolonged grooming duration (Figure 1H) following water spray compared to controls (Ctrl), indicating succinate‐associated compulsive‐like behavior. No significant differences were observed between SA and Ctrl mice in body weight (Figure S1A,B), water consumption (Figure S1C, D), locomotion activity (Figure 1B), or anxiety‐like behavior (Figure 1C–E), confirming that the compulsive‐like phenotype in succinate‐treated mice was independent of body weight, water intake, or anxiety levels. Machine learning‐assisted quantification of spontaneous behavior revealed distinctive ethograms in SA mice versus Ctrl via principal component analysis (PCA) of movement features (Figure 1K). Specifically, SA mice exhibited reduced exploration behaviors (including hunching and rearing) and significantly increased maintenance behaviors (including grooming) (Figure 1I,J). Kinematic parameters remained unchanged (Figure S1E), aligning with unaltered locomotion in the open field test (Figure 1B).

Given our prior identification of *Parabacteroides distasonis* (*P. distasonis*) enrichment in feces of mice transplanted from drug‐naïve OCD patients [12], we next tested whether this succinate‐producing bacterium alone could contribute to compulsive‐like behavior. Antibiotic‐pretreated mice inoculated with *P. distasonis* (P.dis) (Figure 1L) exhibited significantly elevated anxiety‐like (Figure 1O,P) and compulsive‐like (Figure 1Q–S) behaviors relative to vehicle‐treated (Veh) controls. Collectively, these data indicate an association between gut‐derived succinate and excessive compulsive‐like behaviors.

### 2.2 Hypoactivity of dmPFC Glutamatergic Neurons Underlies Succinate‐Associated Compulsive‐Like Behaviors

Given the established role of the dorsal medial prefrontal cortex (dmPFC) in the pathophysiology of OCD [18], and our prior findings demonstrating that dmPFC activity regulates OCD patient FMT‐associated compulsive‐like behaviors [12], we hypothesized that dmPFC glutamatergic neurons modulate succinate‐associated compulsive‐like phenotypes.

**FIGURE 2:**
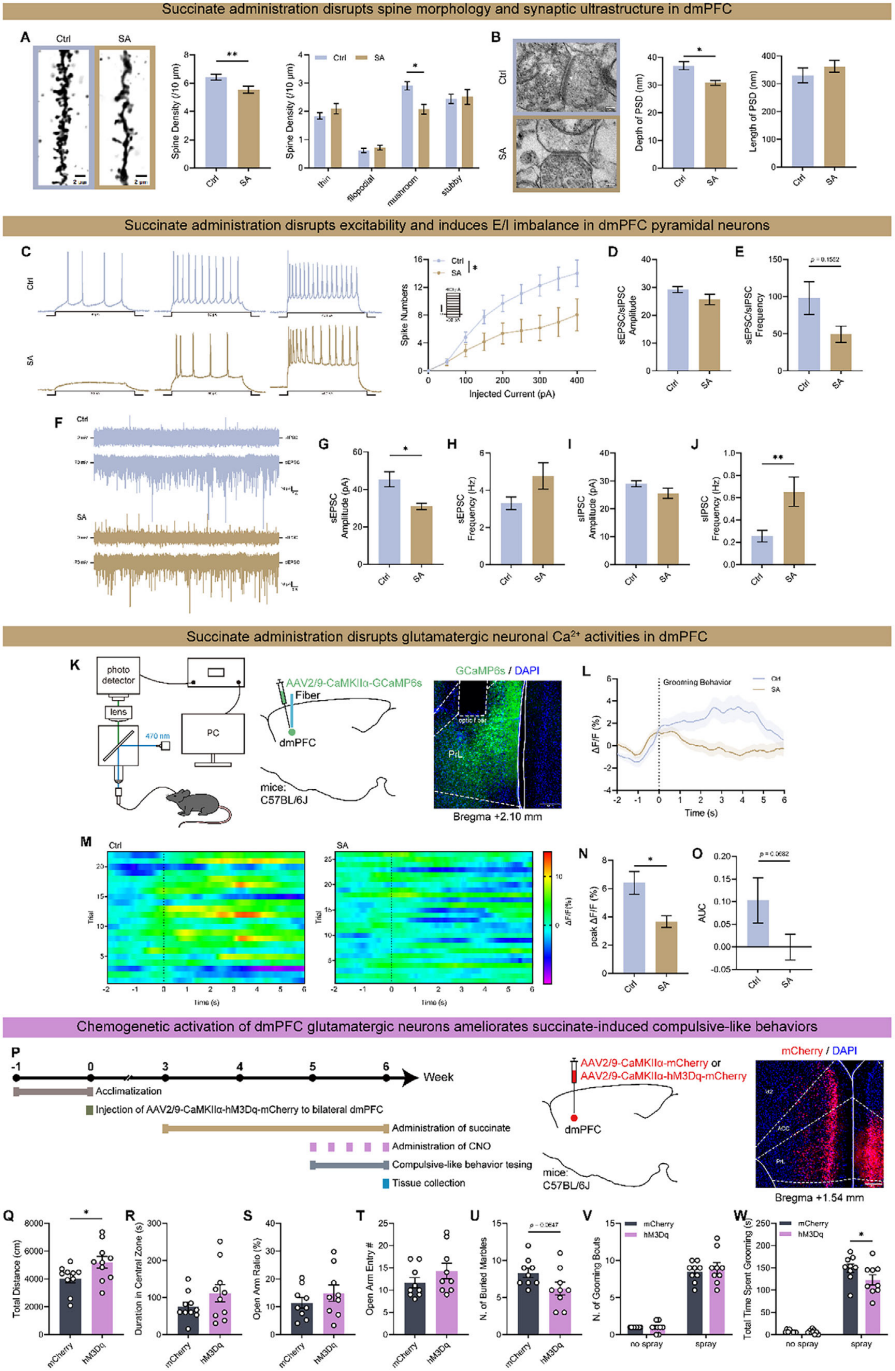
Hypoexcitability of dmPFC glutamatergic neurons mediates succinate‐associated compulsive‐like behavior. (A) Left: Representative images of spines in the dmPFC. Middle: Spine density in the dmPFC. Right: Density of each spine type in the dmPFC. (*n* = 38 images from 4 Ctrl mice, *n* = 26 images from 3 SA mice). (B) Left: Representative images of PSD ultrastructure in the dmPFC. Middle: Metrics of PSD ultrastructure assessed by depth. Right: Metrics of PSD ultrastructure assessed by length. (*n* = 19 images from 3 Ctrl mice, *n* = 32 images from 3 SA mice). (C) Left: Representative traces of action potentials of dmPFC pyramidal neurons. Right: Spike numbers of action potentials of dmPFC pyramidal neurons. (*n* = 16 neurons from 3 Ctrl mice, *n* = 24 neurons from 4 SA mice). (D and E) sEPSC/sIPSC amplitude ratio (D) and sEPSC/sIPSC frequency ratio (E) of dmPFC pyramidal neurons. (*n* = 13 neurons from 3 Ctrl mice, *n* = 8 neurons from 3 SA mice). (F–J) Representative traces (F), sEPSC amplitude (G), sEPSC frequency (H), sIPSC amplitude (I), and sIPSC frequency (J) of dmPFC pyramidal neurons. (*n* = 13 neurons from 3 Ctrl mice, *n* = 8 neurons from 3 SA mice). (K) Left: Schematic of fiber photometry setup. Middle: Schematic of glutamatergic neuron‐specific GCaMP6s in the dmPFC, and fiber photometry recording. Right: Representative image showing GCaMP6s expression and optic fiber cannula placement in the dmPFC. (L–O) Heatmap (M), average ΔF/F ratio (L), peak ΔF/F (N), and AUC (O) of dmPFC glutamatergic neuron calcium signals aligned to grooming onset. (*n* = 22 trials from 4 Ctrl mice, *n* = 26 trials from 5 SA mice). (P) Left: Experimental design for assessing the effects of activating dmPFC glutamatergic neurons on succinate‐associated compulsive‐like behaviors. Middle: Schematic of glutamatergic neuron‐specific hM3Dq‐mCherry in the dmPFC. Right: Representative image showing mCherry expression in the dmPFC. (Q‐T) Locomotor activity and anxiety‐like behaviors assessed by total distance traveled in the open field (Q), time spent in the central zone (R), proportion of time spent in the open arms (S), and frequency of entries into the open arms (T). (*n* = 9 mCherry, *n* = 9 hM3Dq). (U–W) Compulsive‐like behaviors assessed by number of buried marbles (U), number of grooming bouts (V), and total grooming duration (W). (*n* = 9 mCherry, *n* = 9 hM3Dq). Each dot represents an individual mouse. Data are represented as mean ± SEM. Statistical significance was determined by unpaired two‐tailed *t*‐test (Q–U), nested *t*‐test (A, B, D, E, G–J, N, O), or two‐way ANOVA (C, V, W). *p < 0.05; **p < 0.01; ***p < 0.001; ****p < 0.0001.

To determine whether succinate administration affects dendritic spines in the dmPFC, the functional sites of synaptic transmission [19], we performed Golgi‐Cox staining [20]. We assessed spine characteristic, including classification into morphological subtypes (thin, filopodial, mushroom, and stubby) indicative of dendritic spine maturation status [21, 22]. The results showed a significant decrease in total dendritic spine density in SA mice compared to Ctrl mice (Figure 2A). Analysis of morphological subtypes further revealed that the density of mushroom‐type spines was significantly reduced in the SA mice relative to Ctrl mice (Figure 2A). These findings suggest that succinate exposure may influences dmPFC neuroplasticity, potentially through the diminution of mature spines.

To determine whether succinate administration impact neural ultrastructure in the dmPFC, we performed transmission electron microscopy (TEM) to assess synaptic and myelin integrity. TEM analysis revealed that SA mice exhibited significantly thinner postsynaptic density (PSD) (Figure 2B) and reduced myelin sheath thickness (Figure S2A–D) compared to the Ctrl group. These ultrastructural disruptions suggest potential impairments in the electrophysiological properties of dmPFC neurons.

To further investigate whether succinate exposure is associated with altered neuronal function in the dmPFC, we conducted whole‐cell patch‐clamp recordings on dmPFC pyramidal neurons. Pyramidal neurons from SA mice displayed a significant reduction in the number of action potentials elicited by current injection compared to Ctrl neurons (Figure 2C). Additionally, SA mice exhibited decreased spontaneous excitatory postsynaptic current (sEPSC) amplitude (Figure 2G) and increased spontaneous inhibitory postsynaptic current (sIPSC) frequency (Figure 2J) in dmPFC pyramidal neurons. Consequently, the sEPSC/sIPSC frequency ratio (Figure 2E) was reduced in SA mice. These results indicate that succinate administration is associated with neuronal hypoactivity and an altered excitatory/inhibitory (E/I) balance within dmPFC pyramidal neurons.

We next employed fiber photometry to directly assess in vivo alterations in the activity of dmPFC glutamatergic neurons following succinate administration (Figure 2K). GCaMP6s‐expressing dmPFC glutamatergic neurons in SA mice showed significantly reduced peak calcium activity and diminished area under the curve (AUC) of calcium transients during grooming bouts (Figure 2L–O) and exploratory behaviors (Figure S2E–H) compared to controls. These in vivo measurements of diminished neuronal activity further corroborate the association between succinate exposure and reduced electrophysiological function of dmPFC glutamatergic neurons.

Having established the detrimental effects of succinate exposure on dmPFC neuronal structure and function, we next examined whether activation of dmPFC glutamatergic neurons could rescue succinate‐associated compulsive‐like behaviors. Using chemogenetic techniques, we selectively activated hM3Dq‐expressing dmPFC glutamatergic neurons during behavioral testing via intraperitoneal injection of clozapine‐N‐oxide (CNO, 2 mg/kg) (Figure 2P). CNO injection successfully activated dmPFC neurons (Figure S2I), and importantly, activation of dmPFC glutamatergic neurons significantly ameliorated the compulsive‐like behaviors following succinate administration (Figure 2U–W).

Collectively, these results demonstrate that hypoactivity of dmPFC glutamatergic neurons underlies succinate‐associated compulsive‐like behaviors.

### 2.3 Succinate Exposure Is Associated With Altered dmPFC Microglial Transcriptomic Profiles and Microglial Hyperactivation

**FIGURE 3:**
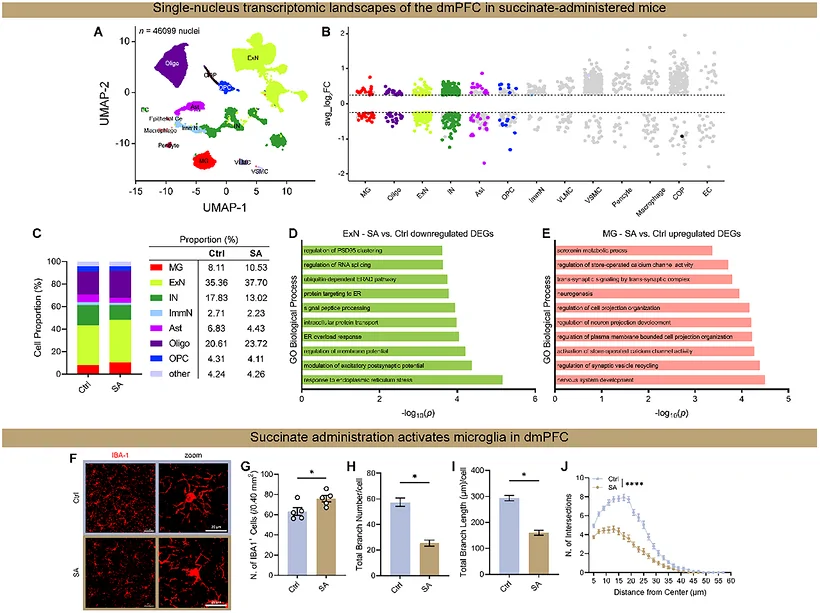
Succinate exposure is associated with microglia altered transcriptomic profiles and hyperactivation in the dmPFC. (A) Uniform manifold approximation and projections (UMAP) plot of single‐nuclei from all combined samples, colored by annotated cell type. (B) Strip chart showing DEGs across cell types. Colored dots represent DEGs with adjusted *p* < 0.05, and grey dots represent DEGs with *p* < 0.05 but adjusted *p* > 0.05. (C) Composition plot and table showing cell type distribution per experimental condition. (D) Significantly top enriched GO biological processes for downregulated DEGs in excitatory neurons of SA mice. (E) Significantly top enriched GO biological processes for upregulated DEGs in microglia of SA mice. (F,G) Representative image (F) and density (G) of IBA‐1 positive microglia in the dmPFC. (*n* = 5 Ctrl, *n* = 5 SA). (H–J) Morphology metrics of microglia indicated by total branch number (H), total branch length (I), and Sholl analysis results (J). (*n* = 51 cells from 5 Ctrl mice, *n* = 47 cells from 5 SA mice). Each dot represents an individual mouse. Data are represented as mean ± SEM. Statistical significance was determined by unpaired two‐tailed *t*‐test (G), nested *t*‐test (H, I), or two‐way ANOVA (J). *p < 0.05; **p < 0.01; ***p < 0.001; ****p < 0.0001.

To investigate whether succinate exposure is associated with altered transcriptomic profiles within the dmPFC, we performed single‐nucleus RNA sequencing (snRNA‐seq) on dmPFC tissue from Ctrl and SA mice. We profiled a total of 46 099 nuclei. Unsupervised clustering analysis identified 35 transcriptionally distinct cell clusters (Figure S3A), subsequently annotated as 14 major cell types (Figure 3A) based on canonical marker gene expression (Figure S3D). Comparative analysis revealed significant alterations in cellular composition within the dmPFC of SA mice compared to Ctrl. Notably, the proportion of microglia was significantly elevated in SA mice (Figure 3C). Differential expressed gene (DEGs) analysis (Figure 3B) of excitatory neurons and subsequent pathway enrichment revealed that succinate treatment downregulated biological processes for synaptic transmission, including modulation of excitatory postsynaptic potential, regulation of membrane potential, and regulation of postsynaptic density protein 95 clustering in the excitatory neurons (Figure 3D). These transcriptomic alterations align with the spine loss, disrupted PSD ultrastructure, and electrophysiological dysfunction we observed in dmPFC glutamatergic neurons. Furthermore, we observed significant downregulation of microglial homeostatic markers, including *Tmem199* and *Hexb* [23], in the dmPFC of succinate‐treated mice (Table S1), suggesting succinate exposure‐associated disruption of microglial homeostasis and promotion of a pathological state. Enrichment analysis of DEGs in microglia indicated upregulation of biological processes related to the regulation of neurons and synapses in SA mice (Figure 3E), implying a potential role for microglia in modulating neural activity and synaptic transmission under conditions of succinate exposure.

To convince microglia activation status in the dmPFC, we performed immunofluorescence imaging using the microglia‐specific marker ionized calcium‐binding adapter molecule 1 (IBA‐1) (Figure 3F). The density of microglia was significantly higher in the dmPFC of SA mice compared to Ctrl mice (Figure 3G). Furthermore, microglia in the dmPFC of SA mice exhibited a less complex morphology than those in Ctrl mice, as indicated by reduced branch number (Figure 3H), decreased total branch length (Figure 3I), and fewer intersections with Sholl rings (Figure 3J). These morphological alterations collectively indicate an association between succinate administration and microglial hyperactivation in the dmPFC.

### 2.4 Microglial SUCNR1 Mediates Succinate‐Associated Compulsive‐Like Behaviors and Dysfunction of dmPFC Microglia and Glutamatergic Neurons

**FIGURE 4:**
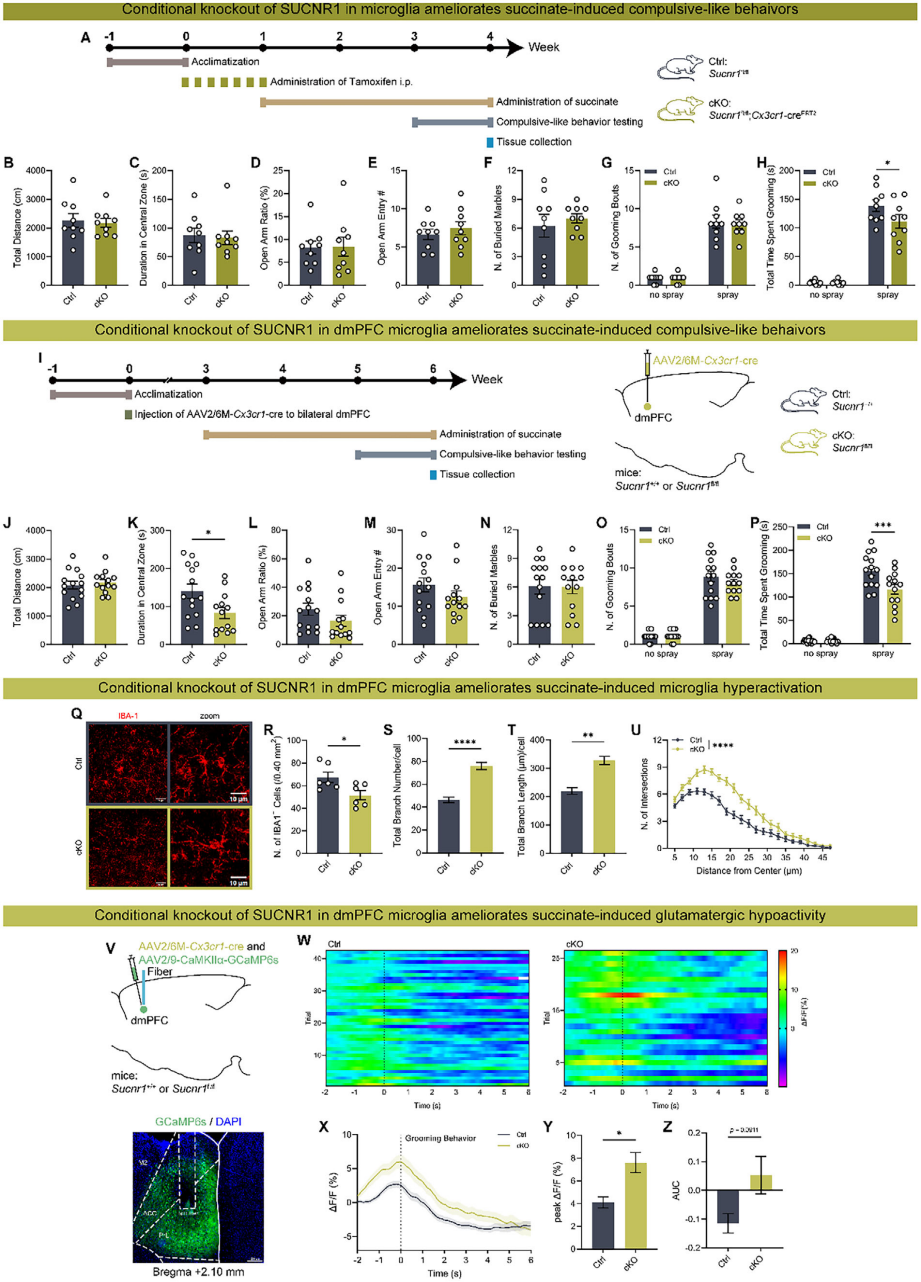
Microglial SUCNR1 is required for succinate‐associated compulsive‐like behaviors and dysfunction of microglia and glutamatergic neurons. (A) Experimental design for assessing the effects of microglia‐specific conditional knockout of SUCNR1 on succinate‐associated compulsive‐like behaviors. (B–E) Locomotor activity and anxiety‐like behaviors assessed by total distance traveled in the open field (B), time spent in the central zone (C), proportion of time spent in the open arms (D), and frequency of entries into the open arms (E). (*n* = 9 Ctrl, *n* = 9 cKO). (F–H) Compulsive‐like behaviors assessed by number of buried marbles (F), number of grooming bouts (G), and total grooming duration (H). (*n* = 9 Ctrl, *n* = 9 cKO). (I) Experimental design for assessing the effects of dmPFC microglia‐specific conditional knockout of SUCNR1 on succinate‐associated compulsive‐like behaviors. (J‐M) Locomotor activity and anxiety‐like behaviors assessed by total distance traveled in the open field (J), time spent in the central zone (K), proportion of time spent in the open arms (L), and frequency of entries into the open arms (M). (*n* = 14 Ctrl, *n* = 13 cKO). (N‐P) Compulsive‐like behaviors assessed by number of buried marbles (N), number of grooming bouts (O), and total grooming duration (P). (*n* = 14 Ctrl, *n* = 13 cKO). (Q and R) Representative image (Q) and density (R) of IBA‐1 positive microglia in the dmPFC. (*n* = 6 Ctrl, *n* = 6 cKO). (S‐U) Morphology metrics of microglia indicated by total branch number (S), total bran length (T), and Sholl analysis results (U). (*n* = 36 cells from 6 Ctrl mice, *n* = 33 cells from 6 cKO mice). (V) Upper: Schematic of microglia‐specific Cre and glutamatergic neuron‐specific GCaMP6s in the dmPFC, and fiber photometry recording. Lower: Representative image showing GCaMP6s expression and optic fiber cannula placement in the dmPFC. (W‐Z) Heatmap (W), average ΔF/F ratio (X), peak ΔF/F (Y), and AUC (Z) of dmPFC glutamatergic neuron calcium signals aligned to grooming onset. (*n* = 42 trials from 8 Ctrl mice, *n* = 26 trials from 7 cKO mice). Each dot represents an individual mouse. Data are represented as mean ± SEM. Statistical significance was determined by unpaired two‐tailed *t*‐test (B, D, E, F, J, K, R), Mann‐Whitney test (C, L, M, N), nested *t*‐test (S, T, Y, Z), or two‐way ANOVA (G, H, O, P, U). *p < 0.05; **p < 0.01; ***p < 0.001; ****p < 0.0001.

A previous study [24] and our own experiments (Figure S4A–E) demonstrated that orally administered ^13^C_2_‐labeled succinate was detectable in the brain. As mice were transcardially perfused with saline before brain collection, and no ^13^C_2_‐succinate signal was observed in saline‐gavage control mice, the labeled succinate measured in brain tissue reflects genuine blood‐to‐brain transfer rather than residual blood contamination. These results indicate that gut‐derived succinate can cross the blood‐brain barrier and enter the CNS. Given our findings that succinate exposure associated with microglial hyperactivation in the dmPFC (Figure 3F–J), coupled with the fact that microglia are the primary macrophage population resident within the CNS [25], along with the established immunomodulatory effect of succinate on macrophages mediated by its cognate receptor SUCNR1 [26], we hypothesize that succinate‐associated activation of dmPFC microglia is mediated by microglial SUCNR1. To test this hypothesis, we crossed *Sucnr1* ^fl/fl^ mice with *Cx3cr1*‐cre^ERT2^ mice to conditionally knockout (cKO) *Sucnr1* expression specifically in microglia (Figure 4A). Tamoxifen was administered prior to succinate treatment to induce *Sucnr1* cKO. Following succinate administration, we demonstrated that microglial *Sucnr1* cKO significantly ameliorated the succinate‐associated compulsive‐like behaviors (Figure 4H), suggesting a modulatory role of microglial SUCNR1 in these behavioral responses.

To further investigate whether SUCNR1 specifically within dmPFC microglia is responsible for succinate‐associated compulsive‐like behavior, we transfected AAV2/6M‐*Cx3cr1*‐cre into the dmPFC of *Sucnr1* ^fl/fl^ mice to achieve region‐specific microglial depletion of *Sucnr1* (Figure 4I). Behavioral analyses revealed that dmPFC microglia‐specific *Sucnr1* cKO successfully ameliorated the succinate‐associated compulsive‐like behavior (Figure 4P), further confirming the mediating role of dmPFC microglial SUCNR1 in this phenotype.

To determine whether microglial *Sucnr1* cKO reversed succinate‐associated microglial hyperactivation, we performed morphological analyses of microglia in the dmPFC (Figure 4Q). Compared to Ctrl mice, *Sucnr1* cKO mice exhibited a significant decrease in the density of IBA‐1‐positive microglia (Figure 4R) along with significant increases in microglial branch number (Figure 4S), total branch length (Figure 4T), and Sholl ring intersections (Figure 4U). These findings indicate that microglial SUCNR1 mediates succinate‐associated hyperactivation within the dmPFC.

Fiber photometry was subsequently employed to assess whether microglial *Sucnr1* cKO could rescue succinate‐associated hypoactivity in dmPFC glutamatergic neurons (Figure 4V). Correspondingly, the peak ΔF/F of calcium transients during grooming bouts were significantly elevated in mice with dmPFC microglial *Sucnr1* cKO compared to control mice (Figure 4W–Z), demonstrating that microglial SUCNR1 mediates the dysfunction of glutamatergic neurons.

Collectively, our serial conditional knockout experiments systematically verified that microglial SUCNR1 mediates succinate‐associated dmPFC microglial and glutamatergic neuronal dysfunction, as well as the manifestation of abnormal compulsive‐like behavior.

### 2.5 Vagus‐DVC‐CeA‐dmPFC Circuit Mediates Succinate‐Associated Compulsive‐Like Behaviors

**FIGURE 5:**
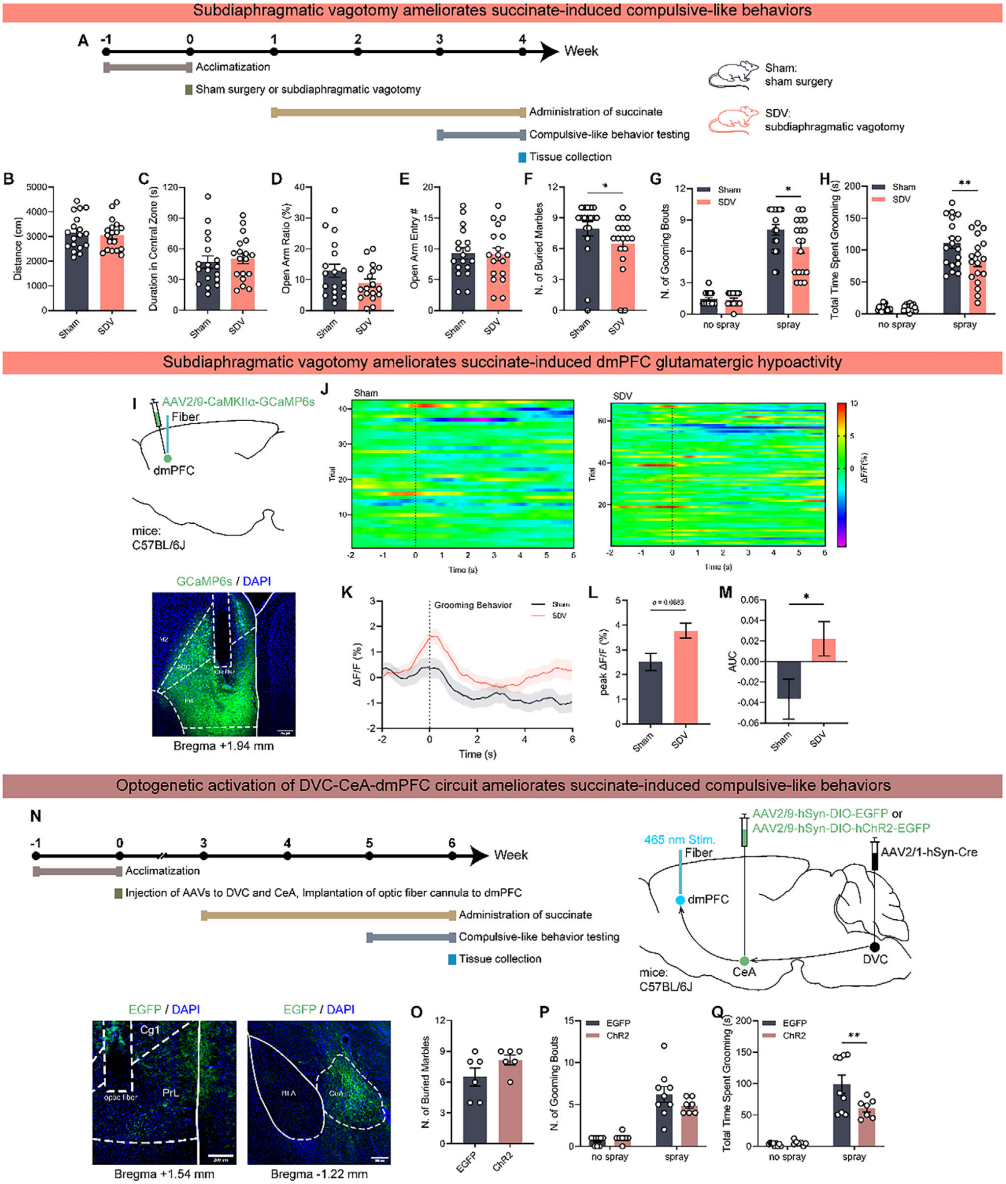
Vagus nerve and DVC‐CeA‐dmPFC circuit mediates succinate‐associated compulsive‐like behavior. (A) Experimental design for assessing the effects of vagus nerve on succinate‐associated compulsive‐like behaviors. (B–E) Locomotor activity and anxiety‐like behaviors assessed by total distance traveled in the open field (B), time spent in the central zone (C), proportion of time spent in the open arms (D), and frequency of entries into the open arms (E). (*n* = 16 Sham, *n* = 18 SDV). (F–H) Compulsive‐like behaviors assessed by number of buried marbles (F), number of grooming bouts (G), and total grooming duration (H). (*n* = 16 Sham, *n* = 18 SDV). (I) Upper: Schematic of glutamatergic neuron‐specific GCaMP6s in the dmPFC, and fiber photometry recording. Lower: Representative image showing GCaMP6s expression and optic fiber cannula placement in the dmPFC. (J–M) Heatmap (J), average ΔF/F ratio (K), peak ΔF/F (L), and AUC (M) of dmPFC glutamatergic neuron calcium signals aligned to grooming onset. (*n* = 42 trials from 5 Sham mice, *n* = 68 trials from 5 SDV mice). (N) Upper left: Experimental design for assessing the effects of DVC‐CeA‐dmPFC circuit on succinate‐associated compulsive‐like behaviors. Upper right: Schematic of viral strategy for optogenetic activation of DVC‐CeA‐dmPFC circuit. Lower: Representative image showing EGFP expression and optic fiber cannula placement in the dmPFC, and EGFP expression in the CeA. (O–Q) Compulsive‐like behaviors assessed by number of buried marbles (O), number of grooming bouts (P), and total grooming duration (Q). (MBT: *n* = 6 EGFP, *n* = 6 ChR2; groom: *n* = 9 EGFP, *n* = 7 ChR2). Each dot represents an individual mouse. Data are represented as mean ± SEM. Statistical significance was determined by unpaired two‐tailed *t*‐test (B, C, E), Mann‐Whitney test (D, F), nested *t*‐test (L, M), or two‐way ANOVA (G, H, P, Q). *p < 0.05; **p < 0.01; ***p < 0.001; ****p < 0.0001.

Beyond the CNS alterations demonstrated above, we investigated the contribution of the peripheral nervous system to succinate‐associated behavioral abnormalities. Given that the vagus nerve serves as a major conduit for gut‐brain communication [16], we performed subdiaphragmatic vagotomy (SDV) to assess whether gut‐derived succinate also influences the central nervous system via this pathway. Serial behavioral testing revealed that SDV effectively ameliorated succinate‐associated compulsive‐like behavior (Figure 5F–H), suggesting that vagal signaling transmits succinate‐related information from the gut to the brain.

To further determine whether the vagus nerve mediates succinate‐associated hypoactivity of glutamatergic neurons in the dmPFC, we employed fiber photometry in SDV‐operated mice expressing GCaMP6s in dmPFC glutamatergic neurons (Figure 5I). Compared to sham‐operated controls, AUC of calcium signal aligned with grooming behavior were significantly elevated in SDV mice (Figure 5J–M). These results demonstrate that vagal innervation is essential for succinate‐associated behavioral abnormalities and dmPFC glutamatergic neuronal hypoactivity.

Next, we sought to delineate the neural circuit through which the vagus nerve conveys gut‐derived signals to the dmPFC. Based on prior literature [27, 28] and our anterograde tracing data following intestinal injection of herpes simplex virus (HSV), which showed tdTomato‐labeled fluorescence signals in the dorsal vagal complex (DVC)—predominantly within the dorsal motor nucleus of the vagus (DMV) (Figure S5F), we hypothesized that the DVC serves as the primary central relay receiving vagal afferents from the gut. As the DVC lacks direct projections to the dmPFC [28], we focused on the central amygdala (CeA) as a potential intermediary node, given its connectivity from the DVC [29] and projections to the mPFC. Using optogenetics, we selectively stimulated axon terminals of the DVC‐CeA pathway within the dmPFC. Activation of this DVC‐CeA‐dmPFC circuit partially rescued the compulsive‐like behavior in succinate‐administered mice (Figure 5Q).

Collectively, our findings identify a vagus nerve‐mediated gut‐to‐brain pathway (vagus‐DVC‐CeA‐dmPFC) that underlies compulsive‐like behaviors associated with succinate exposure.

### 2.6 Gut 5‐HT_4_ Receptor Mediates Succinate‐Associated Compulsive‐Like Behaviors

**FIGURE 6:**
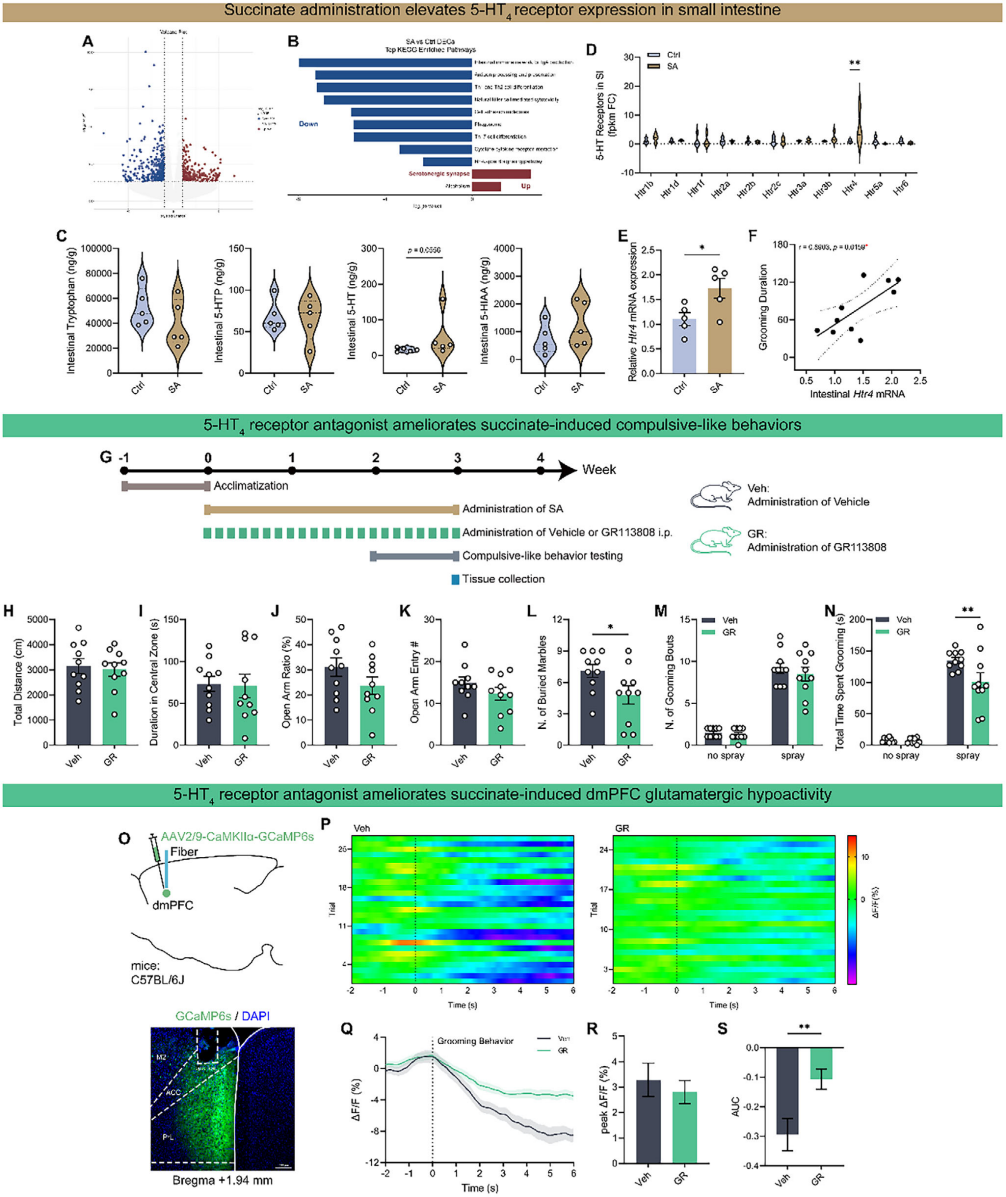
5‐HT_4_ receptor mediates succinate exposure‐associated compulsive‐like behavior. (A) Volcano plots showing DEGs in the small intestine of SA mice versus Ctrl mice. (B) Top‐enriched KEGG pathways of DEGs in the small intestine of SA mice versus Ctrl mice. (C) Quantification of the intestinal metabolites involving in serotonergic synapse pathway in normalized levels. (*n* = 5 Ctrl, *n* = 5 SA). (D) Expression of 5‐HT receptor mRNA in the small intestine. (*n* = 4 Ctrl, *n* = 4 SA). (E) RT‐qPCR validation of the expression of *Htr4* in the small intestine. (*n* = 5 Ctrl, *n* = 5 SA). (F) Correlation between intestinal *Htr4* mRNA expression and grooming duration after water spray. (*n* = 5 Ctrl, *n* = 5 SA). (G) Experimental design for assessing the effects of 5‐HT_4_ receptor on succinate‐associated compulsive‐like behaviors. (H–K) Locomotor activity and anxiety‐like behaviors assessed by total distance traveled in the open field (H), time spent in the central zone (I), proportion of time spent in the open arms (J), and frequency of entries into the open arms (K). (*n* = 10 Veh, *n* = 10 GR). (L–N) Compulsive‐like behaviors assessed by number of buried marbles (L), number of grooming bouts (M), and total grooming duration (N). (*n* = 10 Veh, *n* = 10 GR). (O) Upper: Schematic of glutamatergic neuron‐specific GCaMP6s in the dmPFC, and fiber photometry recording. Lower: Representative image showing GCaMP6s expression and optic fiber cannula placement in the dmPFC. (P‐S) Heatmap (P), average ΔF/F ratio (Q), peak ΔF/F (R), and AUC (S) of dmPFC glutamatergic neuron calcium signals aligned to grooming onset. (*n* = 27 trials from 8 Veh mice, *n* = 26 trials from 9 GR mice). Each dot represents an individual mouse. Data are represented as mean ± SEM. Statistical significance was determined by unpaired two‐tailed *t*‐test (C, E, H–L), nested *t*‐test (R, S), or two‐way ANOVA (D, M, N). *p < 0.05; **p < 0.01; ***p < 0.001; ****p < 0.0001.

Following the establishment of the peripheral vagus nerve's role in succinate‐associated compulsive‐like behavior and its underlying neural circuitry, we sought to elucidate the molecular mechanisms through which gut‐derived signals modulate vagal activity to impact calcium dynamics in dmPFC glutamatergic neurons, thereby mediating succinate exposure‐associated behavioral pathology. RNA sequencing and untargeted metabolomics were performed on intestinal tissue, revealing transcriptomic (Figure S6A) and metabolic (Figure S6D) profiles between SA and Ctrl mice. Since no metabolites reached statistical significance based on the criteria of FDR‐adjusted *p* < 0.05 in the untargeted metabolomics screening, the intestinal untargeted metabolomic dataset was used solely for discovery purposes. Gene Ontology (GO) enrichment analysis of DEGs (Figure 6A) showed that downregulated DEGs were enriched in immune‐related GO terms (Figure S6B), whereas upregulated DEGs were associated with synaptic function and cell division (Figure S6C). KEGG pathway analysis further identified the serotonergic synapse signaling pathway as the most significantly enriched pathway among up‐regulated DEGs (Figure 6B). Furthermore, the serotonergic synapse signaling pathway emerged as the overlapping pathway co‐enriched in both DEGs and DAMs (Figure S6E). To validate the metabolic alterations related to this pathway in intestinal tissue, we performed targeted quantification of relevant metabolites in an independent batch of samples. Notably, serotonin, the key metabolite in the serotonergic synapse pathway, was marginally elevated in the intestinal tissue of the SA group (Figure 6C), and was significantly correlated with the grooming behavior of mice after water spray (Figure S6F,G). Given the centrality of serotonin receptors in serotonergic synapses, we assessed the FPKM expression levels of these receptors in the small intestine. Among them, only *Htr4* (encoding the 5‐HT_4_ receptor) mRNA expression was significantly altered in SA mice (Figure 6D), a result that was confirmed by RT‐qPCR in a separate batch of samples (Figure 6E). Moreover, intestinal *Htr4* mRNA expression was significantly correlated with the grooming duration after water spray (Figure 6F), while fecal succinate concentration showed a marginal correlation with the intestinal *Htr4* mRNA expression (Figure S6H).

As 5‐HT_4_ receptor activation modulates vagal excitability [30, 31, 32], we administered the peripheral antagonist GR113808 [33] to determine whether 5‐HT_4_ receptor inhibition ameliorates succinate‐associated compulsive‐like behavior (Figure 6G). GR113808 significantly attenuated behavioral abnormalities (Figure 6L–N), confirming the receptor's mediatory role. Fiber photometry further demonstrated that 5‐HT_4_ receptor inhibition rescues succinate‐associated hypoactivity in dmPFC glutamatergic neurons (Figure 6O–Q). The AUC of calcium transients in GCaMP6s‐expressing neurons was significantly elevated in GR113808‐treated SA mice (Figure 6S), indicating partial reversal of succinate's detrimental effects on neuronal electrophysiology.

Collectively, these results demonstrate that succinate administration reprograms intestinal transcriptomic and metabolic landscapes, and that 5‐HT_4_ receptors critically mediate succinate‐associated compulsive‐like behaviors via modulation of dmPFC glutamatergic neuronal activity.

### 2.7 Targeting dmPFC Succinate Metabolism Ameliorates Compulsive‐Like Behaviors in *Hoxb8* ^−/−^ Mice

**FIGURE 7:**
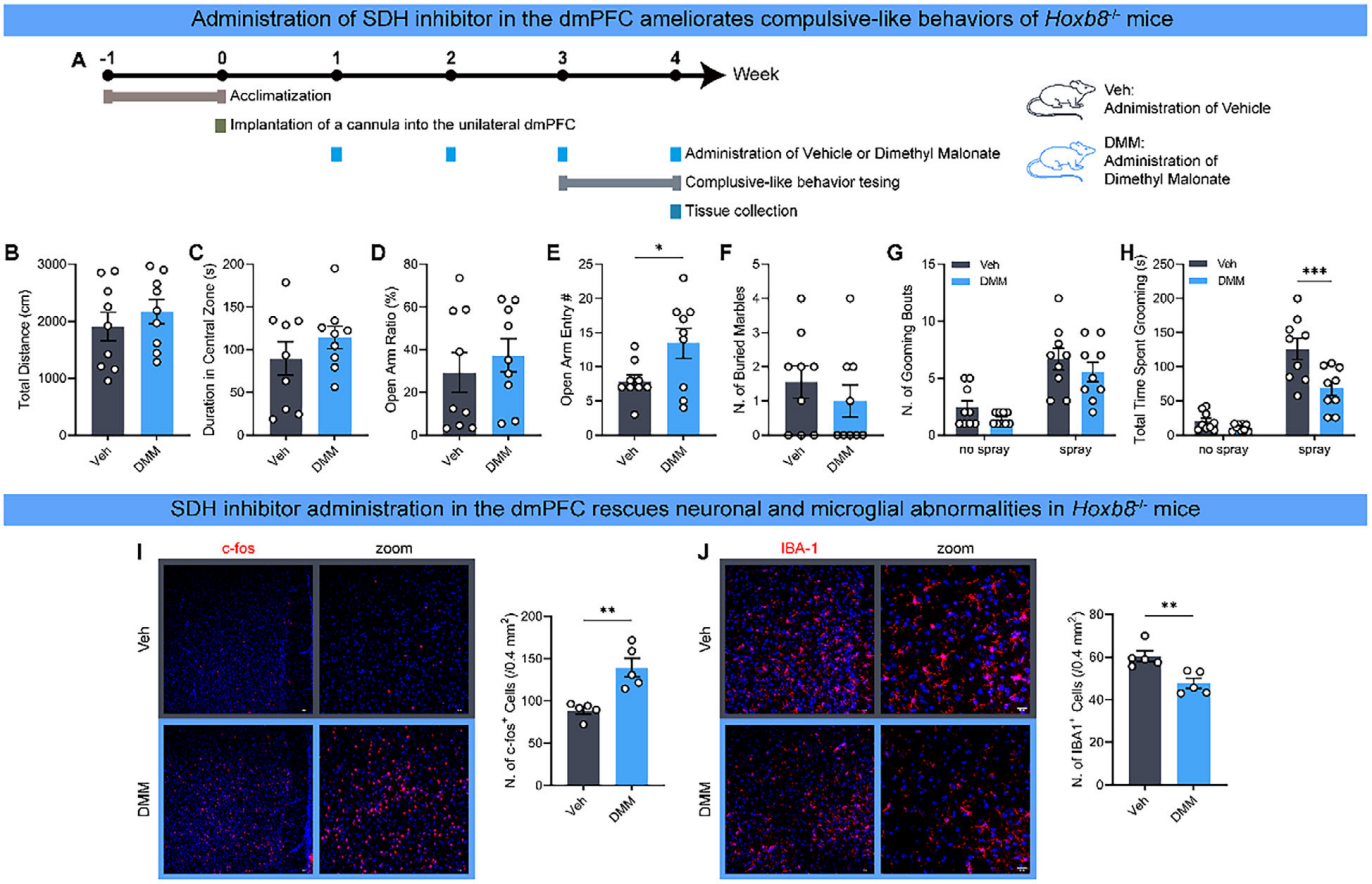
Inhibiting SDH in dmPFC ameliorates compulsive‐like behaviors of *Hoxb8* ^−/−^ mice. (A) Experimental design for assessing the effects of SDH inhibitor on compulsive‐like behaviors of *Hoxb8* ^−/−^ mice. (B–E) Locomotor activity and anxiety‐like behaviors assessed by total distance traveled in the open field (B), time spent in the central zone (C), proportion of time spent in the open arms (D), and frequency of entries into the open arms (E). (*n* = 9 Veh, *n* = 9 DMM). (F‐H) Compulsive‐like behaviors assessed by number of buried marbles (F), number of grooming bouts (G), and total grooming duration (H). (*n* = 9 Veh, *n* = 9 DMM). (I) Left: Representative images of c‐fos^+^ activated cells in the dmPFC. Right: Number of c‐fos^+^ cells in the dmPFC of *Hoxb8* ^−/−^ mice. (*n* = 5 Veh, *n* = 5 DMM). (J) Left: Representative images of IBA‐1^+^ cells in the dmPFC. Right: Number of IBA‐1^+^ microglia in the dmPFC of *Hoxb8* ^−/−^ mice. (*n* = 5 Veh, *n* = 5 DMM). Each dot represents an individual mouse. Data are represented as mean ± SEM. Statistical significance was determined by unpaired two‐tailed *t*‐test (B, C, E, I, J), Mann‐Whitney test (D, F), or two‐way ANOVA (G, H). *p < 0.05; **p < 0.01; ***p < 0.001; ****p < 0.0001.

Succinate dehydrogenase (SDH), an enzyme complex catalyzing a key step in both the tricarboxylic acid cycle and oxidative phosphorylation [34], mediates the immunomodulatory functions of succinate [13, 35]. To evaluate the therapeutic potential of targeting SDH‐related succinate metabolism pathway for OCD, we administered dimethyl malonate (DMM), a competitive inhibitor of SDH [35, 36], into the dmPFC of *Hoxb8* ^−/−^ mice. This transgenic line is a well‐established model of trichotillomania, an OCD‐spectrum disorder, exhibiting excessive anxiety and compulsive‐like behaviors [37, 38]. Weekly administration of DMM into the dmPFC significantly ameliorated anxiety‐like (Figure 7E) and compulsive‐like (Figure 7H) behaviors of *Hoxb8* ^−/−^ mice, demonstrating the therapeutic efficacy of targeting the succinate metabolism pathway. Immunofluorescence analysis further confirmed that SDH inhibition increased neuronal activation (as indicated by c‐fos positive cells; Figure 7I) and reduced microglial numbers (Figure 7J) within the dmPFC of *Hoxb8* ^−/−^ mice.

Collectively, these findings indicate that SDH inhibition ameliorates excessive compulsive‐like behavior in *Hoxb8* ^−/−^ mice, potentially by mitigating neuronal and microglial dysfunction in the dmPFC.

### 2.8 Succinate is Associated With Functional Activity in the Prefrontal Subregions of OCD Patients

Building on our preclinical findings, we conducted new analyses in our well‐established cohort [39] to further assess the clinical relevance and translational potential of serum succinate in OCD. Although the initial finding of increased serum succinate and its correlation with symptom severity were reported in this cohort previously [12], we performed receiver operating characteristic (ROC) analysis to evaluate its diagnostic potential here for the first time. The ROC curve (Figure S7A) yielded an AUC of 0.73 (95% CI: 0.61 – 0.85), indicating a moderate discriminatory power of serum succinate for distinguishing patients with OCD from healthy controls.

Given that succinate modulates neuronal excitability in the PFC in mice, we further examined the amplitude of low‐frequency fluctuation (ALFF), fractional ALFF (fALFF), and regional homogeneity (ReHo) derived from resting‐state functional MRI (rs‐fMRI) data in our established OCD cohort [39] to investigate potential correlations between serum succinate levels and PFC functional activity. Serum succinic acid level in OCD patients was found significantly correlated with zfALFF of Frontal_Sup_R, Frontal_Mid_L, and Frontal_Mid_R (Figure S7B–D, Table S3). These results indicate that elevated serum succinate in OCD patients may mediate functional activity alterations within specific prefrontal subfields, thereby influencing OCD symptom severity.

## 3 Discussion

Accumulating evidence demonstrates that gut microbiota and their derived metabolites play pivotal roles in the pathophysiology of psychiatric disorders. Building upon our prior findings suggesting a potential pathogenic contribution of succinate to OCD [12], this study elucidates the precise mechanisms underlying succinate‐associated compulsive‐like behavioral abnormalities. We identified a critical role for gut‐brain axis signaling in mediating these effects. Specifically, we found that hypofunction of glutamatergic neurons within the dmPFC serves as a central mediator of the aberrant behavior. Succinate‐associated alterations in dmPFC glutamatergic neuronal function involve two pathways: SUCNR1‐mediated microglia hyperfunction within the dmPFC, and signal transmission along the vagus nerve to downstream neural circuits. Furthermore, pharmacological targeting of succinate metabolism effectively rescued behavioral deficits of *Hoxb8* ^−/−^ mice, highlighting the therapeutic promise of modulating succinate signaling pathways for OCD and its related disorders.

### 3.1 dmPFC Glutamatergic Neurons Mediate Succinate‐Associated Compulsive‐Like Behavior

As a core component of the cortico‐striato‐thalamo‐cortical (CSTC) circuit hypothesis, the prefrontal cortex (PFC) is established as a critical mediator in the pathophysiology of OCD [40, 41, 42, 43]. Clinical studies consistently demonstrate structural and functional abnormalities within the PFC in OCD patients [18, 44], and non‐invasive brain stimulation techniques targeting this region [45, 46, 47, 48] show efficacy in reducing symptom severity. While preclinical evidence implicates the PFC in pathological behavioral abnormalities in OCD rodent models [43, 49, 50], including the regulation of excessive self‐grooming behaviors [51, 52, 53], a translationally relevant analogue of compulsive‐like behavior [54], detailed mechanistic insights into its specific dysfunction and causal role in driving pathological compulsive‐like behavior remain incompletely understood. Our study specifically elucidates the critical role of dmPFC glutamatergic neurons in driving pathological compulsive‐like behavior and identifies underlying structural and electrophysiological deficits. Using multimodal analyses—including spine morphology, PSD ultrastructure, patch‐clamp electrophysiology, and fiber photometry—we demonstrate a reduction in mature dendritic spines, disrupted PSD architecture, and postsynaptic excitatory hypoactivity in dmPFC glutamatergic neurons. Critically, successful rescue of succinate‐associated compulsive‐like behavior via selective chemogenetic activation of dmPFC glutamatergic neurons underscores the potential of targeting this specific circuit node as a therapeutic strategy for OCD. This finding aligns with clinical evidence demonstrating that excitatory stimulation of the PFC significantly improves OCD symptoms [45].

### 3.2 Microglial SUCNR1 Mediates Succinate‐Associated Compulsive‐Like Behavior

Succinate, functioning as an immunometabolite [13, 15], engages its cognate receptor SUCNR1 to modulate immune functions in specific cell types, including macrophages [26]. The impact of the succinate‐SUCNR1 signaling axis on macrophages exhibits significant heterogeneity across distinct pathological contexts and tissue types, necessitating context‐specific evaluation of its functional consequences [26]. While the succinate‐SUCNR1 pathway has been implicated in the regulation of several peripheral physiological and pathological processes [55], its role within the CNS remains poorly defined. Given that SUCNR1 is predominantly expressed on microglia, the brain's resident macrophages [25, 56], we specifically targeted microglial SUCNR1 to investigate its contribution to succinate exposure‐associated neuronal dysfunction and behavioral abnormalities. Utilizing a microglia‐specific conditional knockout strategy, we observed a significant attenuation of succinate‐associated compulsive‐like behaviors. Morphological analysis of microglia further revealed that SUCNR1 ablation ameliorated succinate‐associated hyperactivation, reinforcing the pathway's significance in brain pathophysiology. Considering the pivotal role of microglia‐neuron crosstalk [56, 57], we employed fiber photometry and demonstrated that microglial SUCNR1 conditional knockout successfully rescued the succinate‐associated hypoactivity of glutamatergic neurons within the dmPFC. Collectively, these findings provide compelling evidence for a critical role of microglial SUCNR1 in mediating succinate exposure‐associated neuropathological and behavioral phenotypes.

Notably, while immune dysregulation and microglial involvement have been implicated in the pathogenesis of OCD [58, 59], the precise mechanistic contributions of microglia—particularly through immunometabolic pathways—remain poorly explored. Our study not only provides direct experimental evidence supporting microglial dysfunction in succinate‐associated compulsive‐like behaviors, but also establishes the succinate‐SUCNR1 axis on microglia as a novel and targetable pathway for developing precision therapeutic strategies against neuropsychiatric disorders characterized by metabolic‐inflammatory crosstalk.

### 3.3 A Gut‐to‐brain Circuit Linking Gut to dmPFC Modulates Succinate‐Associated Compulsive‐Like Behavior

Vagal sensory afferents transduce gut‐derived metabolite signals to the DVC within the brainstem, which subsequently modulates downstream neural circuits [16, 60]. Employing subdiaphragmatic vagotomy, we established that vagal afferents convey succinate signals from the gut to suppress activity in dmPFC glutamatergic neurons, thereby mediating succinate‐associated compulsive‐like behaviors. Given the absence of direct DVC‐dmPFC projections [28], we postulated the existence of a polysynaptic DVC→CeA→dmPFC circuit based on published data [29] and validated its functional role in mediating succinate's effects using projection‐specific optogenetic manipulation. To elucidate the peripheral mechanism underlying succinate‐vagal signaling, we performed RNA‐seq coupled with RT‐qPCR on small intestinal tissue. This approach identified the 5‐HT_4_ receptor as a putative mediator, consistent with prior reports implicating this receptor in vagal activity modulation [30, 31, 32]. Pharmacological antagonism of the 5‐HT_4_ receptor ameliorated succinate‐associated compulsive‐like phenotypes and hypoactivity of dmPFC glutamatergic neurons. Collectively, these findings delineate a gut‐brain axis wherein vagal‐mediated succinate signaling links to dmPFC neuronal hypoactivity via a DVC‐CeA‐dmPFC circuit. Furthermore, intestinal 5‐HT_4_ receptor upregulation is identified as a critical transducer within this metabolite‐to‐neural signaling pathway.

### 3.4 Targeting Succinate‐Related Pathway: Therapeutic Potential for OCD

SSRIs and CBT, particularly exposure and response prevention, constitute the first‐line treatment for OCD [61]. However, a substantial proportion of patients remain refractory to current therapeutic modalities, failing to achieve adequate symptom relief or remission [3]. Our study proposes several novel therapeutic strategies targeting key components of the succinate metabolic and signaling pathway: antagonism of the succinate receptor SUCNR1, blockade of the 5‐HT_4_ receptor, and inhibition of SDH. The structural characterization of SUCNR1 [55] and the succinate‐SUCNR1 binding mechanism [62, 63] are well‐established. Potent human‐selective antagonists, such as NF‐56‐EJ40 [64], have demonstrated therapeutic efficacy in human‐derived disease models (e.g., clear cell renal carcinoma [65]). While these findings underscore the therapeutic promise of SUCNR1 antagonism, the current absence of SUCNR1 antagonists capable of binding rodent SUCNR1 [62] hinders their application in validated preclinical rodent models of OCD for efficacy assessment in alleviating compulsive‐like behavioral abnormalities. Rigorous evaluation in alternative validated models, such as human patient‐derived organoids, could therefore provide critical insights to further elucidate the relevance of SUCNR1 targeting for OCD therapy. Given the distribution of 5‐HT_4_ receptors in both peripheral tissues and the central nervous system [66], and our focus on the role of peripheral 5‐HT_4_ receptors in succinate‐associated compulsive‐like behaviors, future preclinical studies should utilize peripherally restricted 5‐HT_4_ receptor antagonists incapable of crossing the blood‐brain barrier. This approach is critical to mitigate potential confounding neuropsychiatric side effects arising from central 5‐HT_4_ receptor inhibition. Collectively, our findings illuminate the succinate signaling axis as a promising therapeutic target for the development of innovative interventions for treatment‐resistant OCD.

### 3.5 Limitations of the Study

While our study delineates the underlying molecular and circuit mechanisms by which succinate exposure associates with compulsive‐like behavioral abnormalities and provides a foundation for developing novel therapies targeting succinate signaling for OCD, several limitations warrant acknowledgments. First, the specific contributions of inhibitory neurons and local microcircuits within the dmPFC, as well as the roles of downstream circuits receiving dmPFC projections, in succinate‐associated compulsive‐like behaviors remain to be elucidated. Future studies are needed to dissect the comprehensive circuit mechanisms underlying this succinate exposure‐associated effects. Second, although we employed fiber photometry to assess the impact of microglial SUCNR1 on calcium activity in dmPFC glutamatergic neurons, further experiments are required to elucidate the precise molecular mechanisms mediating microglia‐neuron interactions and the resulting modulation of glutamatergic neuronal properties. This mechanistic insight is crucial to substantiate our bioinformatic findings derived from snRNA‐seq data. Third, the casual relationship between intestinal mucosal succinate exposure and the upregulation of *Htr4* mRNA expression was inferential. The detailed mechanisms for succinate to upregulate intestinal 5‐HT_4_ receptor, and targeted deletion of 5‐HT_4_ receptor specifically in vagal afferent neurons is necessary to conclusively determine the role of vagal 5‐HT_4_ receptor signaling in succinate's effects and its downstream consequences. Additionally, future investigations should address whether combinatorial targeting of microglial SUCNR1 and ablation of either gut 5‐HT_4_ receptor of vagal afferent activity exerts additive or synergistic ameliorative effects on succinate‐associated compulsive‐like behaviors. Moreover, our study only employed male mice. Given the well‐documented sexual dimorphism in gut microbiota composition, serotonergic signaling, and microglia properties, as well as our initial finding that succinate may act as a potential pathological metabolite was derived exclusively from a male cohort, it was reasonable to focus on male mice in the present investigation. The robust behavioral phenotype observed in males allowed us to dissect the underlying biological mechanisms with fewer confounding factors and greater clarity. Future studies are needed to extend these results to female mice, encompassing both behavioral phenotypes and the underlying biological mechanisms, in order to fully characterize the sex‐specific nature of the observed effects. Future studies should also employ more refined behavioral paradigms and cross‐species translational approaches to better recapitulate the complex manifestations of psychiatric disorders.

Nevertheless, our study establishes a solid foundation for future exploration into the translational potential of mentioned target toward novel therapeutic strategies for OCD.

## 4 Conclusion

In conclusion, our research demonstrates that gut‐derived succinate and succinate‐producing *P. distasonis* are associated with significant compulsive‐like behavioral phenotypes in mice. We identify two distinct signaling pathways linked to this behavioral phenotype: microglial SUCNR1 activation and vagal afferent fiber signaling. Both pathways impair the function of dmPFC glutamatergic neurons, which is in turn linked to succinate‐associated compulsive‐like behavior. These findings underscore the critical impact of a gut‐derived pathological metabolite in CNS function via the gut‐brain axis and provide mechanistic insights for novel therapeutic strategies targeting OCD. Further investigations should evaluate the translational relevance of the proposed therapeutic targets for precision medicine.

## 5 Methods

### 5.1 Human

A total of 32 drug‐naïve OCD patients were recruited at the Shanghai Mental Health Center between January 2021 and October 2022 from an existing clinical cohort enrolled in a study registered at ClinicalTrials.gov (Clinical Study Registration Number: NCT04539951), the design of which has been described previously [39]. Key inclusion criteria comprised: age 18–65 years; a primary diagnosis of OCD meeting Diagnostic and Statistical Manual of Mental Disorders, Fifth Edition (DSM‐5) criteria; a Yale‐Brown Obsessive‐Compulsive Scale (Y‐BOCS) score ≥ 24; no prior pharmacotherapy for OCD; no OCD‐specific psychotherapy within the preceding 6 months; and provision of written informed consent. Key exclusion criteria included: meeting the DSM‐5 diagnostic criteria for schizophrenia spectrum and other psychotic disorders, or bipolar and related disorders; moderate to severe suicide ideation or suicide attempts; severe depression (Beck Depression Inventory‐II score ≥ 29); comorbid psychiatric or medical disorders potentially impacting metabolite measurements; and pregnancy or lactation. Additionally, 32 gender‐, age‐, and education level‐matched healthy controls (HC) were recruited via community advertisements. HC eligibility required no personal or family history of psychiatric or neurological disorders, as confirmed by the Mini‐International Neuropsychiatric Interview (MINI). All participants (OCD and HC) had no history of substance or alcohol abuse, significant physical illness, or traumatic brain injury. The study protocol was reviewed and approved by the Ethics Committee of the Shanghai Mental Health Center (2020‐11C4). Written informed consent was obtained from all participants prior to their enrollment.

The human serum succinate data, including the group comparisons and their correlation with clinical symptom severity, were originally reported in our prior study [12]. The present analyses were performed on baseline samples and data collected from this registered cohort (registration number: NCT04539951), and these data were subjected to novel and hypothesis‐driven analyses. Specifically, the ROC analysis evaluating the diagnostic potential of serum succinate, and the correlation analyses between serum succinate levels and rs‐fMRI metrics, are presented here for the first time and constitute novel contributions of this work. The details of these analytical methods are described in the respective sections below.

### 5.2 Animals

Wild‐type C57BL6/J mice were purchased from Vital River, GemPharmatech, or Cyagen Biosciences. *Sucnr1* ^fl/fl^, *Sucnr1* ^fl/fl^;*Cx3cr1*‐cre^ERT2^, and *Hoxb8* ^−/−^ mice were purchased from Cyagen Biosciences. All male mice used in this study were over 8 weeks of age. Mice were group‐housed (3–5 per cage) under a 12‐h light/dark cycle with food and water ad libitum at the specific pathogen‐free (SPF) animal research centers of Shanghai Jiao Tong University or Shanghai Mental Health Center. All animal experimental procedures were performed in accordance with institutional guidelines and approved by the Institutional Animal Care and Use Committee (IACUC) of Shanghai Jiao Tong University (A2024338‐001).

### 5.3 Administration of Succinate

Oral administration of sodium succinate for modeling lasted at least 2 weeks. During this period, water bottles and their contents in each cage were refreshed every other day. Control group mice received sterilized water without sodium succinate. For the sodium succinate administration group, sodium succinate was dissolved in sterilized water at a concentration of 15 mg/mL.

### 5.4 Administration of *Parabacteroides. distasonis* (*P. distasonis*)

Prior to *P. distasonis* inoculation, mice received an antibiotic cocktail in their drinking water for 2 weeks to deplete the gut microbiota. The antibiotic cocktail consisted of: ampicillin (1 mg/mL; Sangon Biotech), metronidazole (1 mg/mL; Sangon Biotech), neomycin (1 mg/mL; Sangon Biotech), vancomycin (0.5 mg/mL; Sangon Biotech) and saccharin (4 mg/mL; Sangon Biotech). Saccharin was added to mask the taste of the antibiotics. The antibiotic solution was replaced every other day to prevent degradation.

*P. distasonis* (ATCC 8503) was cultured and propagated in trypticase soy agar/broth supplemented with defibrinated sheep blood (BNCC359963, BeNa Culture Collection) according to ATCC protocols. Mice received oral gavage of *P. distasonis* (10^6^ colony‐forming units [CFU] per 200 µL suspended in sterile anaerobic PBS) every other day for 2 weeks. Control mice received gavage of sterile anaerobic PBS at the same volume (200 µL).

### 5.5 Administration of Drugs

For chemogenetic manipulation of dmPFC glutamatergic neuronal activity using clozapine N‐oxide (CNO), mice in both the mCherry and hM3Dq groups received intraperitoneal injections of CNO (100 µL, 2 mg/kg) approximately 30 min prior to behavioral testing. The CNO working solution was prepared by dissolving CNO in saline.

To induce conditional knockdown of *Sucnr1* in microglia of *Sucnr1* ^fl/fl^;*Cx3cr1*‐cre^ERT2^ mice, both *Sucnr1* ^fl/fl^ control and *Sucnr1* ^fl/fl^;*Cx3cr1*‐cre^ERT2^ mice received intraperitoneal injections of tamoxifen (100 µL, 40 mg/kg) once daily for six consecutive days. The tamoxifen working solution was prepared by dissolving tamoxifen in 90% corn oil and 10% ethanol. Subsequent experiments commenced one week after the final tamoxifen administration.

For pharmacological inactivation of the 5‐HT_4_ receptor using GR113808, the GR group received intraperitoneal injections of GR113808 (200 µL, 1 mg/kg) once daily for a minimum of two consecutive weeks. The GR113808 working solution was prepared by dissolving GR113808 in saline containing 1% DMSO. GR113808 administration was initiated concurrently with sodium succinate treatment and ceased upon its completion. As a vehicle control (Veh group), mice received i.p. injections of vehicle solution (200 µL; saline containing 1% DMSO) at the same frequency and duration as the GR group.

For dimethyl malonate microinjection into the dmPFC, the DMM group received weekly microinjections of dimethyl malonate (0.2 µL, 15 µg/µL) for four consecutive weeks. The dimethyl malonate working solution was prepared by dissolving dimethyl malonate in saline. Microinjection was performed at a flow rate of 1 nL/s. As a vehicle control (Veh group), mice received microinjections of saline (0.2 µL) at the same frequency and duration as the DMM group.

### 5.6 Behavior Measurement

All behavioral testing was conducted during the light phase under dim lighting conditions. Prior to experimentation, mice were acclimated to the behavioral room for at least 1 h. To eliminate residual scent cues between trials, apparatus surfaces were cleaned with 75% ethanol after each mouse completed testing. Behavioral outcomes were scored by observers blinded to experimental conditions.

#### 5.6.1 Open Field Test (OFT)

Mouse activity was measured in an open field arena (40 cm × 40 cm × 40 cm, central zone: 20 cm × 20 cm) for 10 min. Metrics included the total distance traveled in the open field arena, and the duration spent in the central zone, to assess locomotor activity and anxiety‐like behavior, respectively.

#### 5.6.2 Elevated Plus Maze Test (EPM)

The EPM consisted of two opposing open arms (30 cm × 5 cm) and two opposing closed arms (30 cm × 5 cm), elevated above the floor. Each mouse was placed in the central zone facing an open arm away from the experimenter at the start of the 5‐min trial. Mice falling off the maze during testing were excluded. Metrics included open arm ratio [calculated as time spent in open arms / (time spent in open arms + time spent in closed arms)], and number of open arm entries. Both metrics serve as indices of anxiety‐like behavior.

#### 5.6.3 Marble Buried Test (MBT)

Prior to the beginning of the trial, corncob bedding (5 cm depth) was evenly distributed in the cage. 12 glass marbles were then placed on the bedding surface in a 3 × 4 grid (4 cm apart). Each mouse was introduced into the cage for 30 min. The metric was the number of marbles buried to at least 2/3 of their depth in the bedding.

#### 5.6.4 Grooming Behavior Test

Mice were individually placed in cages without bedding. Following a 5‐min period, grooming was induced by delivering 4 water sprays near the head. Grooming behavior, defined as face wiping, whole‐body grooming, or head/ear scratching, was recorded. The frequency and duration of grooming bouts were quantified during the 5‐min periods before and after water spraying.

#### 5.6.5 Spontaneous Behavior Analysis

Spontaneous mouse behavior was recorded using BehaviorAtlas 3D‐AI, a multi‐view video capture system. 4 cameras were orthogonally mounted on the supporting pillars of the apparatus. Mice were individually placed into a circular open field arena (50 cm diameter, 50 cm height) for a 5‐min session.

For spontaneous behavior analysis, the BehaviorAtlas Analyzer software was employed to automatically classify behavioral phenotypes into 40 distinct clusters. Subsequently, the BehaviorAtlas Explorer software was used to randomly sample and inspect at least 70% of the video clips for each behavioral cluster to identify movement phenotypes and group them by shared behavioral significance. Manual adjustment and refinement of labels for specific segments were performed to enhance movement classification accuracy. The 40 behavior clusters were ultimately categorized into three broad categories: Locomotion (running, trotting, walking, left turning, right turning), Exploration (jumping, rearing, hunching, rising, sniffing, upstretching), and Maintenance (grooming, ticking) [67]. For kinematic parameter assessment, 16 body points were tracked: nose, left ear, right ear, neck, left front limb, right front limb, left hind limb, right hind limb, left front claw, right front claw, left hind claw, right hind claw, back, root tail, mid tail, and tip tail.

### 5.7 Golgi‐Cox Staining

Golgi‐Cox staining was performed using a commercial kit (Saint‐Bio). Whole brains were fixed and subsequently immersed in the staining solution at room temperature for a minimum of 2 weeks, protected from light. Coronal sections (200 µm thickness) were prepared using a vibratome (VT1000s, Leica, Germany) and processed according to the manufacturer's protocol. Stained sections were imaged using a super‐resolution microscope system (IXplore IX83 SpinSR, Olympus). Spine density (expressed as the number of spines per 10 µm dendritic segment) and the relative proportion of each spine morphological subtype (classified as thin, filopodial, mushroom, or stubby) within the total spine population were quantified using ImageJ.

### 5.8 Transmission Electronic Microscopy

Following saline perfusion, mice were perfused with electron microscopy fixative (2.5% glutaraldehyde in PBS). Brain tissue was rapidly excised, and the target region was dissected into 1 mm^3^ pieces. Tissue pieces were fixed in the same fixative at 4°C for a minimum of 6 h prior to further processing. Subsequent preparation steps comprised: rinsing in 0.1 M PB (4 rounds, 10 min each), post‐fixation in 1% osmium tetroxide (0.2 mL, 2 h), additional PB rinses (3 rounds, 10 min each), and dehydration through a graded ethanol series (30%, 50%, 70%, 90%; 15 min each). Tissues were then infiltrated with acetone mixtures (90% ethanol:90% acetone, 1:1 for 20 min; 90% acetone for 20 min), followed by dehydration in 100% acetone (3 rounds, 10 min each). Resin infiltration was performed stepwise using acetone:resin mixtures (1:1 for 1 h, 1:2 for 2 h, 1:3 overnight). After overnight infiltration, resin was refreshed twice the following day. Samples were embedded in resin within molds and polymerized at 60°C for 48 h. Ultrathin sections (60 nm) were cut using an ultramicrotome (EM UC7, Leica, Germany), stained with uranyl acetate and lead citrate, and imaged using a transmission electron microscope (Tecnai G2, Spirit Biotwin).

For ultrastructural analysis, the following metrics were quantified: postsynaptic density (PSD) thickness and length, and myelin sheath thickness. The g‐ratio, defined as the axon diameter divided by the diameter of the entire myelinated fiber, was calculated for myelin assessment.

### 5.9 Electrophysiological Recording

Mice were anesthetized with isoflurane and transcardially perfused with ice‐cold cutting solution containing 212 mM sucrose, 3 mM KCl, 125 mM NaH_2_PO_4_, 26 mM NaHCO_3_, 10 mM D‐glucose, and 7 mM MgCl_2_·6H_2_O. Following decapitation, brains were rapidly removed and coronal sections (300 µm thick) were prepared using a vibratome (VT1000s, Leica, Germany) while submerged in oxygenated (95% O_2_, 5% CO_2_) cutting solution. Targeted slices were incubated in oxygenated (95% O_2_, 5% CO_2_) artificial cerebrospinal fluid (aCSF; containing 125 mM NaCl, 2.5 mM KCl, 1.25 mM NaH_2_PO_4_, 1.3 mM MgSO_4_, 26 mM NaHCO_3_, 2 mM CaCL_2_·2H_2_O, and 10 mM D‐glucose) at 31°C for 30 min, followed by 60 min equilibration at room temperature. Prepared slices were transferred to a recording chamber continuously perfused (approximately 4 mL/min) with oxygenated aCSF at room temperature during recordings. Neurons were visualized using infrared differential interference contrast microscopy (U‐TLUIR, Olympus). Whole‐cell patch‐clamp recordings were obtained using a Multiclamp 700B amplifier (Molecular Devices). Borosilicate glass pipettes (BF150‐86‐10, Sutter Instrument) were pulled (P‐97, Sutter Instrument) before use, yielding resistances of 2–5 MΩ to optimize seal quality.

For current‐clamp recordings, the pipette solution contained 130 mM K‐gluconate, 5 mM KCl, 2.5 mM MgCl_2_, 4 mM Na_2_ATP, 0.4 mM Na_3_GTP, 10 mM Na phosphocreatine, 10 mM HEPES, and 0.5 mM EGTA. Signals were filtered at 2.5 kHz and sampled at 5 kHz. Neuronal excitability was assessed by injecting depolarizing current steps (500 ms duration, 60 s inter‐step interval) from −50 to +400 pA in 50 pA increments.

For voltage‐clamp recordings, the pipette solution contained 115 mM CsMeCO_3_, 20 mM CsCl, 10 mM HEPES, 2.5 mM MgCl_2_, 4 mM Na_2_ATP, 0.4 mM Na_3_GTP, 10 mM Na phosphocreatine, and 0.6 mM EGTA. Signals were filtered at 2.5 kHz and sampled at 10 kHz.

### 5.10 Stereotaxic Surgery

Mice were anaesthetized with isoflurane and secured in a stereotactic frame (RWD Instruments). Head fur was removed, the scalp was disinfected with iodine solution, and a midline incision was made to expose the skull. H_2_O_2_ solution was applied to the skull surface to clearly visualize the bregma. Target brain regions were identified referenced to coordinates defined as anterior‐posterior (AP) from the bregma, mediolateral (ML) from the midline, and dorsal‐ventral (DV) from the brain surface. Viral vectors or cannulas were then injected or implanted into the target regions using the following coordinates: dmPFC: AP = +1.94 mm, ML = 0.37 mm, DV = 1.90 mm; CeA: AP = −1.05 mm, ML = 2.60 mm, DV = −4.95 mm; DVC: AP = −7.20 mm, ML = 0.35 mm, DV = −4.30 mm. Viruses were injected at a volume of 200 nL per site and a flow rate of 1 nL/s using a pulled glass pipette connected to a pressure microinjector (R‐480, RWD Instruments). The pipette remained in place for 10 min post‐injection before withdrawal to minimize fluid reflux. Optic fiber cannulas and guide cannulas were affixed to a holder for implantation and secured to the skull using 2 skull screws and dental cement. After surgery, mice recovered from anesthesia on a heat pad.

For chemogenetic manipulation, AAV2/9‐CaMKIIα‐hM3Dq‐mCherry (5.25 × 10^12^ vg/mL; BrainVTA) or control AAV2/9‐CaMKIIα‐mCherry (5.29 × 10^12^ vg/mL; BrainVTA) was injected bilaterally into the dmPFC. For optogenetic manipulation, AAV2/1‐hSyn‐Cre (1.09 × 10^13^ vg/mL; BrainVTA) was injected unilaterally into the DVC, AAV2/9‐hSyn‐DIO‐hChR2‐EGFP (5.06 × 10^12^ vg/mL; BrainVTA) or control AAV2/9‐hSyn‐DIO‐EGFP (5.28 × 10^12^ vg/mL; BrainVTA) was injected unilaterally into the CeA, and an optic fiber cannula was implanted into the dmPFC. For fiber photometry, AAV2/9‐CaMKIIα‐GCaMP6s (5.25 × 10^12^ vg/mL; BrainVTA) was injected unilaterally into the dmPFC, and an optic fiber cannula was implanted at the injection site. For microglia conditional knockdown, AAV2/6M‐*Cx3cr1*‐Cre (5.88 × 10^12^ vg/mL; BrainVTA) was injected bilaterally into the dmPFC. For microinjection, a guide cannula was implanted unilaterally at the dmPFC.

For experiments involving AAVs, behavioral testing commenced at least 3 weeks post‐injection to allow for sufficient expression. Following all experimental procedures, fluorescent imaging was performed to verify viral expression and cannula placement accuracy. Only data from subjects with confirmed correct targeting were included in the final analysis.

### 5.11 Fiber Photometry

A fiber photometry system (Inper Signal, Inper) was used to record calcium signals from dmPFC glutamatergic neurons. GCaMP fluorescence was elicited using a 470 nm light beam delivered through an optical fiber coupled to the implanted optic fiber cannula (200 µm diameter, 0.37 numerical aperture; Inper). The GCaMP signal was sampled at 40 Hz. To minimize photobleaching, laser intensity was set to 30 µW at the fiber tip. Fiber photometry recordings were synchronized with video recordings of exploratory or grooming behavior.

Fiber photometry data was analyzed using Inper Plot (Inper). GCaMP signals were measured by ΔF/F, where ΔF equaled to F—F_0_. F_0_ was defined as the average baseline fluorescence signal over the 2‐s period before specific behavioral events.

### 5.12 Chemogenetic Manipulation

To manipulate the activity of dmPFC glutamatergic neurons transfected with AAV2/9‐CaMKIIα‐hM3Dq‐mCherry, CNO was administered via intraperitoneal injection. The CNO working solution was prepared in saline. Approximately 30 min prior to behavioral testing, both the mCherry and hM3Dq group received an intraperitoneal injection of 100 µL CNO working solution at a dose of 2 mg/kg.

### 5.13 Optogenetic Manipulation

The implanted optic fiber cannula was connected to a laser generator (Inper). For optogenetic activation of terminals, 465‐nm light beam was delivered through the coupled optical fiber in a setting stimulation paradigm (5 ms pulses, 10 Hz).

### 5.14 Subdiaphragmatic Vagotomy

Mice from both the SDV and Sham groups underwent an overnight fast prior to surgery. Following induction of anesthesia with isoflurane, an approximately 1‐cm incision was made perpendicular to the abdominal midline in the subxiphoid region to expose the upper abdominal organs. A sterile saline‐moistened cotton swab was used to gently push the liver and adjacent viscera into the upper right abdominal quadrant. A glass dissector was then employed to retract and expose the gastroesophageal junction. Under microscopic guidance, the subdiaphragmatic vagal trunks flanking the esophagus were identified and transected bilaterally. In Sham mice, the esophagus was retracted to visualize the vagus nerves, but the trunks remained intact. Subsequent experiments were conducted 1 week postoperatively. Successful vagotomy was confirmed by observing gastric dilation.

### 5.15 Immunofluorescence

Brain tissue was harvested from mice transcardially perfused with saline followed by 4% paraformaldehyde (PFA). The brains were then post‐fixed in PFA, immersed in PBS containing 30% sucrose until sinking, and embedded in optimal cutting temperature (OCT) compound. Coronal sections (30 µm thickness) were prepared using a cryostat (CM1950, Leica). For immunofluorescence staining, sections were heated at 37°C for 2–3 h and rinsed in PBS (2 washes, 3 min each). Sections were blocked with quick blocking buffer for 10 min at room temperature in a humidified chamber and subsequently incubated with primary antibodies overnight at 4°C. Following washing in PBS containing Triton‐X100 (PBST; 5 washes, 5 min each), sections were incubated with species‐specific fluorescent secondary antibodies for 90 min at room temperature. After another series of PBST washes (5 washes, 5 min each), slides were mounted using an anti‐fade mounting medium containing DAPI and stored at 4°C.

The primary antibodies used were: rabbit anti‐c‐Fos (1:2000; ab190289, abcam), mouse anti‐c‐Fos (1:1000; ab208942, abcam), rabbit anti‐Iba1 (1:2000; 019‐19741, Wako), rabbit anti‐mCherry (1:1000; ab167453, abcam), and rabbit anti‐GFP (1:500; ab290, abcam). The secondary antibodies used were: Alexa Fluor 488 anti‐mouse IgG (1:2000; #4408, Cell Signaling Technology), and Alexa Fluor 555 anit‐rabbit IgG (1:2000; #4413, Cell Signaling Technology).

Stained sections were imaged using either a research slide scanner (SLIDEVIEW VS200, Olympus) or confocal laser scanning microscopes (FV1200, Olympus). Fluorescence images were analyzed using ImageJ.

### 5.16 Reverse Transcription Quantitative PCR

Total RNA was isolated from frozen tissue using the RNeasy Mini Kit (74104, Qiagen). RNA concentration and quality was assess using a NanoDrop 2000 spectrophotometer (Thermo Fisher Scientific). Complementary DNA (cDNA) was performed from total RNA using the PrimeScript RT Master Mix (RR036A, TaKaRa). Transcripts quantification was conducted using TB Green Premix Ex Taq II (RR820A, TaKaRa) on a CFX96 Touch Real‐Time PCR Detection System (Bio Rad) or an Applied Biosystems 7500 Real‐Time PCR System (Thermo Fisher Scientific). *Gapdh* served as the reference gene. Relative gene expression levels of target genes were calculated using the 2^‐ΔΔCt^ method relative to the control group.

### 5.17 Single‐Nucleus RNA Sequencing

#### 5.17.1 Single‐Nucleus Isolation and snRNA‐seq

snRNA‐seq was performed by Novogene. Briefly, single‐nucleus suspensions were prepared from fresh‐frozen dmPFC tissues harvested from mice. Nuclei extraction was followed by loading the suspension into Chromium microfluidic chips (10X Genomics) using the 3’ v2 chemistry protocol. Nuclei were barcoded with the Chromium Controller (10X Genomics). Reverse transcription of TNA from barcoded nuclei and library construction were performed using the Chromium Single Cell 3’ Reagent kit (10X Genomics) according to the manufacturer's protocol. Sequencing was performed on an Illumina NovaSeq PE150 platform with standard protocols. Library quality control was performed using fastp (v0.20.0) to generate raw read quality metrics.

#### 5.17.2 Data Preprocessing

Raw sequencing data for each snRNA‐seq library were demultiplexed and aligned to the mouse reference genome (mm10) using CellRanger (v7.0.0) with default parameters to generate cell‐gene count matrices. Potential doublets were identified using Scrublet (v0.2), with cells classified as singlets or doublets based on default parameters. All matrices were processed using Seurat (v5.4.0). Individual Seurat objects were created for each sample. Cells were filtered using the following criteria: minimum genes detected per cell = 200, maximum mitochondrial gene percentage = 15%, and exclusion of Scrublet‐identified doublets. Genes detected in fewer than 5 cells across any sample were removed. Subsequently, objects were normalized, and highly variable genes were identified using the “FindVariableGenes” function (selecting the top 2000 features).

To correct for sample‐level batch effects while preserving biological variation between different treatment groups, samples were first merged into a single Seurat object. The merged object was then normalized, scaled, and principal component analysis (PCA) was performed. Canonical correlation analysis (CCA) was applied for sample integration using the “IntegratedLayers” function. The resulting reduction was sued for downstream analyses.

#### 5.17.3 Dimensionality Reduction, Clustering and Cell‐Type Annotation

PCA was performed on integrated and scaled highly variable genes. A shared nearest neighbor graph was constructed using “FindNeighbors” (dims = 1:30). Significant principal components (PCs) for downstream analysis were determined using the “JackStraw” function following Seurat's standard workflow. Uniform manifold approximation and projection (UMAP) was applied for non‐linear dimensionality reduction based on these significant PCs. Cell clusters were identified using the “FindClusters” function, yielding in 35 distinct clusters. Cluster‐specific marker genes were identified using the “FindAllMarkers” function with the Wilcoxon rank‐sum test. All clusters were manually annotated based on conserved marker gene expression, guided by established cell‐type references.

### 5.18 Bulk RNA Sequencing

Total RNA was extracted using TRIzol reagent (Invitrogen) according to the manufacturer's protocol. RNA concentration and quality were assessed using a NanoDrop 2000 spectrophotometer (Thermo Fisher Scientific), while RNA integrity was evaluated using an Agilent 2100 Bioanalyzer (Agilent Technologies). RNA‐seq libraries were prepared using the VAHTS Universal V6 RNA‐seq Library Prep Kit (Vazyme) following the manufacturer's instructions. Sequencing was performed on an Illumina Novaseq 6000 platform, generating 150‐bp paired‐end reads.

Raw reads in FASTQ format were processed using fastp to remove low‐quality reads, yielding clean reads for subsequent analysis. Clean reads were aligned to reference genome using HISAT2. Gene expression levels were quantified as Fragments Per Kilobase of transcript per Million mapped reads (FPKM), and raw read counts were obtained using HTSeq‐count. Principal component analysis (PCA) was performed using R to assess the reproducibility of biological replicates. Differential expression analysis was conducted using the DESeq2 package in R. Genes meeting the thresholds of *p* < 0.05 and | log_2_(Fold Change) | > 1 were defined as differentially expressed genes (DEGs). Hierarchical clustering analysis of DEGs was performed using R to visualize expression patterns across experimental groups and individual samples. Volcano plots were generated using the ggplot2 package in R to visualize the distribution of DEGs. Gene ontology (GO) and Kyoto encyclopedia of genes and genomes (KEGG) pathway enrichment analyses were performed on DEGs using the hypergeometric test to identify significantly enriched functional terms and signaling pathways. Bar plots or bubble plots representing significantly enriched terms were generated using R.

### 5.19 Metabolomics

#### 5.19.1 Untargeted Metabolomics Profiling

Tissue samples were collected (*n* = 5 per group) and extracted for subsequent metabolomic analysis by investigators blinded to group assignments. Each sample was homogenized in methanol. Following centrifugation, the resulting methanolic supernatant was analyzed using an Agilent 6538 UHD Accurate‐Mass Q‐TOF/MS system at Novogene Co., Ltd. (Beijing, China). Total ion chromatogram (TIC) spectra were acquired in both positive and negative ionization modes. Quality control (QC) samples were prepared by pooling equal volumes of all experimental samples and analyzed before, during and after injection. The first three QC injections were used to monitor instrument stability and equilibrate the chromatographic‐mass spectrometric system, followed by three QC samples for segment scanning to support metabolite identification. QC samples interspersed throughout the run were used to assess system stability over the entire experiment and to perform data quality control. Procedural blank samples were also analyzed to assess background contamination and for subsequent signal subtraction.

Raw mass spectrometry data were preprocessed using XCMS for peak detection, alignment, and quantification. Metabolites were identified by matching accurate mass and the high‐quality secondary spectrum database, allowing for adduct ions and setting mass deviation to 10 ppm. MSI Level 2 identification was utilized in untargeted metabolomics for profiling and relative quantification, as no authentic standards or corresponding standard curves were available. Background signals from blank samples were subtracted, and the remaining quantitative results were normalized to obtain relative peak areas. Metabolites with a coefficient of variation > 30% across QC samples were excluded. The stability of the analytical run was assessed by tight clustering of QC samples in principal component analysis (PCA) space. The final dataset comprised identified metabolites with their relative quantitative values. All data processing steps were implemented under the Linux operating system (CentOS 6.6) using R and Python scripts.

Metabolites annotation was conducted using the KEGG, HMDB and LIPIDMaps database. PCA and Partial least squares‐discriminant analysis (PLS‐DA) were performed. Since none of the metabolites survived FDR correction, metabolites meeting the criteria of variable importance in projection (VIP) > 1.0, *p* < 0.05, and | log_2_(Fold Change) | > 1 were defined as differentially accumulated metabolites (DAMs) for discovery‐oriented screening purposes and downstream pathway analysis.

#### 5.19.2 Targeted Quantification of Serotonin Synapse Pathway Metabolites and Succinate

Targeted quantification of L‐tryptophan, 5‐hydroxytryptophan (5‐HTP), serotonin (5‐HT), 5‐hydroxyindoleacetic acid (5‐HIAA), and succinate was performed using LC‐MS/MS. Serotonin pathway metabolites was performed on an ExionLC AD UHPLC system coupled to a QTRAP 6500+ tandem mass spectrometer (AB SCIEX), while succinate was analyzed on a Vanquish Flex UHPLC system coupled to a TSQ Altis tandem mass spectrometer (Thermo Scientific) at Biozeron Biotechnology Co. Ltd. (Shanghai, China). Both systems were equipped with electrospray ionization sources.

Ileal tissue samples (*n =* 5 per group; approximately 0.02 g) were ground under liquid nitrogen and diluted with ultrapure water by vortexing. A 100 µL aliquot of the diluted homogenate was mixed with 300 µL of ice‐cold 80% methanol containing the isotope‐labeled internal standard 3‐indoleacetic acid‐D4. After vortexing and incubation on ice for 10 min, the mixture was centrifuged at 15 000 rpm for 15 min at 4°C, and the supernatant was injected into the LC‐MS/MS system. Feces samples (*n* = 4 per group; approximately 0.02 g) for succinate analysis were extracted using ice‐cold methanol with ^13^C‐succinate as the internal standard following a validated protocol.

Chromatographic separation was performed on a Waters HSS T3 column (2.1 × 150 mm) maintained at 40°C, with an injection volume of 2 µL. For serotonin metabolites, the mobile phase consisted of 0.1% formic acid and 5 mM ammonium acetate in water (A) and 0.1% formic acid in acetonitrile (B), delivered at 0.30 mL/min, with the following gradient: 0–2 min, 95% A; 4 min, 60% A; 7 min, 30% A; 7.5–9 min, 100% B; 9.1–11 min, 95% A. For succinate, the mobile phase consisted of 0.1% formic acid in water (A) and acetonitrile (B), delivered at 0.40 mL/min, with the following gradient: 0–1 min, 60% A; 3.5 min, 40% A; 8.5 min, 25% A; 11.5–13.5 min, 0% A; 16 min, 60% A. The QTRAP 6500+ mass spectrometer was operated in scheduled multiple‐reaction monitoring (MRM) mode with positive/negative polarity switching. Source parameters were as follows: ion source temperature 550°C, ion spray voltage +4500 V/−4500 V; curtain gas 35 psi; ion source gas 1 (GS1) 50 psi; ion source gas 2 (GS2) 55 psi. The TSQ Altis mass spectrometer was operated in MRM mode with positive/negative polarity switching. Source parameters were as follows: ion source temperature 350°C, ion spray voltage +3500 V/–2500 V; sheath gas 35 psi; auxiliary gas 60 psi; collision gas 1.5 psi. MRM transitions, retention times, declustering potentials, and collision energies for the four analytes are listed in Table S5.

Calibration curves were constructed with solvent‐based calibrators prepared by serial dilution of a mixed standard stock solution in methanol, by plotting the analyte‐to‐internal‐standard peak‐area ratio against the analyte‐to‐internal‐standard concentration ratio, using 1/x^2^ weighting. Calibration was linear over 10—20 000 ng/mL for L‐tryptophan (R^2^ = 0.99327), 50–160 000 ng/mL for 5‐HTP (R^2^ = 0.99299), 1–160 000 ng/mL for 5‐HT (R^2^ = 0.99056) and 5‐HIAA (R^2^ = 0.98990), and 1–5000 ng/mL for succinate (R^2^ = 0.9954). Lower limits of quantification (LLOQs), determined by the signal‐to‐noise method as the lowest concentration yielding a signal‐to‐noise ratio ≥ 10, were 10 ng/mL for L‐tryptophan, 50 ng/mL for 5‐HTP, 1 ng/mL for 5‐HT, 1 ng/mL for 5‐HIAA, and 1 ng/mL for succinate. Limits of detection were not separately reported, as the assay was validated for quantitative application and sensitivity was defined at the LLOQ level. All reported sample concentrations exceeded the respective LLOQs and fell within the validated calibration ranges.

QC samples were interspersed throughout each analytical batch, and the peak‐area ratio of standard to internal standard was used to calculate QC relative standard deviations (RSDs). For the serotonin metabolite assay, all QC RSDs were ≤ 15%. For the succinate assay, the QC RSD was 2.02%. Concentrations were back‐calculated from the calibration curves and are reported as absolute amounts in ng/g of tissue.

### 5.20 In Vivo ^13^C‐succinate Tracing

#### 5.20.1 Administration of ^13^C‐Labeled Succinate

Mice received a single dose of [1,4‐^13^C_2_] disodium succinate (MedChemExpress) at 200 mg/kg (relative quantification cohort; *n* = 3 for vehicle control, *n* = 4 for ^13^C_2_‐succinate) or 100 mg/kg (absolute quantification cohort; *n* = 3 for ^13^C_2_‐succinate) by oral gavage in sterile saline. Vehicle control mice received sterile saline alone. Based on previous literature [24] indicating peak labeled‐succinate availability within approximately 1 h after oral administration, mice were deeply anesthetized at 1 h post‐dose, blood was collected by cardiac puncture, and serum was separated by centrifugation (3000 rpm, 20 min, 4°C). Mice were then transcardially perfused with ice‐cold saline, and the dmPFC was rapidly dissected on ice, snap‐frozen in liquid nitrogen, and stored at −80°C until analysis.

#### 5.20.2 Relative Quantification of ^13^C‐Labeled Succinate Isotopologues

For solid tissue, approximately 25 mg of dmPFC was weighed into a 2.0 mL Eppendorf tube with two steel beads, and 500 µL of extraction solution (methanol:ultrapure water = 4:1, v/v) was added. The mixture was vortexed for 30 s, ground at 35 Hz for 4 min, and sonicated in an ice‐water bath for 5 min; this grinding‐and‐sonication cycle was repeated three times in total. For liquid samples, 100 µL of serum was mixed with 400 µL of methanol, vortexed, sonicated in an ice‐water bath for 15 min. After incubation at −40°C for 1 h, samples were centrifuged at 12 000 rpm and 4°C for 15 min, and the resulting supernatant was transferred to injection vials. Equal volumes of supernatant from all individual samples were pooled to prepare a QC sample, which was analyzed under the same instrumental conditions.

Analysis was performed by Biotree Biotechnology Co. Ltd. (Shanghai, China). Chromatographic separation was performed on an Agilent 1290 Infinity II UHPLC system equipped with an Atlantis Premier BEH Z‐HILIC column (2.1 × 150 mm, 1.7 µm; Waters), and samples were maintained at 6°C in the autosampler tray. The mobile phase consisted of ultrapure water:acetonitrile (90:10, v/v) containing 10 mmol/L ammonium acetate (A) and ultrapure water:acetonitrile (10:90, v/v) containing 10 mmol/L ammonium acetate (B), and the injection volume was 2 µL. Mass spectrometric detection was performed on a Thermo Orbitrap Exploris 120 mass spectrometer (Xcalibur 4.4) with an ESI source operated in positive and negative ion modes with full‐scan MS and data‐dependent MS/MS acquisition. The source parameters were: sheath gas flow 50 Arb, auxiliary gas flow 15 Arb, capillary temperature 320°C; spray voltage 3.8 kV (positive) or −3.4 kV (negative). Full MS scans were acquired at a resolution of 60 000 (FWHM at m/z 200), and MS/MS scans were acquired at a resolution of 15 000 with stepped normalized collision energies of 20/30/40.

Raw data were converted to mzXML format using ProteoWizard and processed with an in‐house R program based on XCMS for feature detection, extraction, alignment, and integration. For succinate, the [M‐H]^−^ ion at m/z 117.0193 and its ^13^C isotopologues (m/z 118.0227 and 119.0261 for M+1 and M+2) were extracted using a mass tolerance of 5 ppm and a retention‐time window of ±0.1 min. Isotopologues peak areas were corrected for natural ^13^C abundance, and the labeling extent of succinate was calculated as the sum of the M+1 and M+2 peak areas divided by the sum of M+0 – M+2. Data are reported as the relative abundances of ^13^C‐labeled succinate isotopologues, expressed as the fraction of ^13^C‐labeled species (M+1 to M+2) relative to the total detected succinate (M+0 to M+2), in serum and dmPFC. Pooled QC samples were injected at regular intervals throughout each analytical batch. The Pearson correlation coefficients among QC samples ranged from 0.99 to 0.996, confirming analytical stability.

#### 5.20.3 Absolute Quantification of ^13^C_2_‐Labeled Succinate

For absolute quantification of ^13^C_2_‐labeled succinate, serum and dmPFC samples were extracted as described in 5.20.2. Analysis was performed on an ACQUITY UPLC Premier system (Waters, Milford, MA, USA) coupled to a Xevo TQ‐Absolute triple quadrupole mass spectrometer (Waters, Milford, MA, USA), performed by Instrumental Analysis Center of Shanghai Jiao Tong University. Chromatographic separation was performed on an ACQUITY UPLC BEH T3 column (2.1 × 100 mm, 1.7 µm; Waters) maintained at 45°C, with an injection volume of 1 µL. The mobile phases consisted of 0.1% formic acid in water (A) and 0.1% formic acid in acetonitrile (B), delivered at 0.40 mL/min with a linear gradient from 1% to 100%.

The mass spectrometer was operated in negative‐ion MRM mode with a scan rate of 0.01 s/scan. Source parameters were: capillary voltage 1 kV; source temperature 150°C, desolvation gas temperature 450°C, desolvation gas flow 900 L/h, cone gas flow 50 L/h. Nitrogen was used as the desolvation and cone gas, and argon as the collision gas. ^13^C_2_‐labeled succinate was monitored using the MRM transitions m/z 119.0 → 74.9 (cone voltage 20 V, collision energy 20 eV).

Calibration curves for ^13^C_2_‐labeled succinate were constructed using solvent‐based external standards over the range 0.1 – 5000 ng/mL, with 1/x weighting. The coefficients of determination (R^2^) were 0.999368 for serum and 0.999359 for brain tissue. QC samples at 10 ng/mL were interspersed throughout each analytical batch, with QC accuracy ranging from 85% to 115% and precision met the predefined acceptance criterion (RSD ≤ 15%). Concentrations were back‐calculated from the calibration curves and are reported as ng/mL for serum and ng/g for brain tissue. Data acquisition and processing were performed using MassLynx 4.2 (Waters).

### 5.21 fMRI

#### 5.21.1 Acquisition

All MRI scans were performed using a 3.0T Siemens Verio scanner. T1‐weighted structural images were acquired using MPRAGE sequence accurate volume of interest (VOI) placement and tissue segmentation. Parameters were as follows: repetition time (TR) = 2300 ms, echo time (TE) = 3.5 ms, flip angle (FA) = 9°, field of view (FoV) = 256 mm, voxel size = 1.0 × 1.0 × 1.0 mm^3^, matrix = 192 × 256. The acquisition parameters of resting‐state functional images were as follows: TR = 2000 ms, TE = 30 ms, FOV = 220 mm, flip angle = 77°, matrix size = 64 × 64, voxel size = 3.0 × 3.0 × 3.0mm^3^, 50 axial slices with a slice thickness of 3 mm and no slice gap, the fMRI scanning lasted for 480 s, and 240 volumes were obtained. None of the participants showed any clinically significant structural abnormalities upon visual inspection. During the entire scanning process, participants were asked to relax and close their eyes, but not fall asleep during scanning.

#### 5.21.2 Preprocessing

The MRI data were first converted from DICOM to the NIfTI format and organized according to the Brain Imaging Data Structure (BIDS) standards using dcm2niix and custom scripts written in Python (version 3.12.7). Functional and structural MRI data preprocessing was performed using the DeepPrep pipeline (version 25.1.1) [68] executed within a Docker container (Docker Desktop 4.44.2). Although the primary focus was on functional data, structural T1‐weighted images were preprocessed within the pipeline to generate the necessary anatomical references (brain masks and cortical surfaces) for functional registration. The functional image preprocessing pipeline included the following steps: (1) head motion correction using MCFLIRT; (2) slice‐timing correction; (3) susceptibility distortion correction (SDC) using acquired field maps; (4) co‐registration to T1w images via boundary‐based registration (bbregister); and (5) spatial normalization to MNI152 space using non‐linear warping. Structural processing was performed automatically within the pipeline.

#### 5.21.3 Postprocessing and Analysis

Post‐processing was performed using Python scripts based on Nilearn. The first 10 volumes were discarded, and high‐motion volumes (FD > 0.5 mm) were interpolated. Participants were excluded if > 50% of volumes exceeded the motion threshold. Nuisance regression was applied using the Friston‐24 motion model, white matter and CSF signals, and linear trends as covariates, followed by band‐pass filtering (0.01 ‐ 0.08 Hz). Based on the processed data, ALFF and fALFF were calculated via Fast Fourier Transform, and ReHo was computed using Kendall's W (cluster size = 27). All metric maps were Z‐standardized. The mean values of these unsmoothed z‐maps (zALFF, zfALFF, zReHo) were extracted using the AAL116 atlases.

### 5.22 Quantification and Statistical Analysis

All data are presented as mean ± SEM. Statistical analyses were performed using Prism 9 (GraphPad Software). The “*n*” value represents the number of mice per experimental group. Normality was assessed using the Shapiro‐Wilk test, and equality of variances was evaluated using Levene's test. Comparisons between two groups were made using an unpaired two‐tailed *t*‐test (for normally distributed data) or the Mann‐Whitney U test (for non‐normally distributed data). A nested *t*‐test was employed for data with a hierarchical structure. For experiments involving two independent variables, significance was assessed using two‐way ANOVA followed by Bonferroni's multiple comparisons test. Results were considered significant at *p* < 0.05. Details of statistical analyses are provided in the figure legends.

Spearman partial correlation analysis was performed to examine the associations of serum succinate level with fMRI metrics in the human cohort. Age, gender, and OCD severity (assessed by Y‐BOCS) were included as covariates. Partial Spearman correlation coefficients (ρ) were calculated, and FDR‐corrected adjusted *p*‐value < 0.05 was considered statistically significant.

## Author Contributions

Z.W., D.‐D.S, Y.L., and Y.‐D.Z. conceptualized the study. Z.W., D.‐D.S, and Y.L. designed experiments. Y.L., and Y.‐D.Z. performed most of the experiments, with the assistance of X.G., F.T., and M.‐Y.D. D.L., J.G., Z.‐F.Z., and Z.‐Y.B. recruited patients. Y.L. analyzed most of the data, with the assistance of Y.‐D.Z. and X.‐Y.W. W.W. analyzed snRNA‐seq data. X.‐Y.W. analyzed spontaneous behavior data. D.L. analyzed human rs‐fMRI data. Y.L. conducted statistical analysis of the data. Y.L. prepared data illustration. Y.L. wrote the original draft of the manuscript, with the assistance of Y.‐D.Z. Z.W. and D.‐D.S reviewed and edited the manuscript. Z.W. and D.‐D.S supervised the study.

## Funding

This work was supported by grants from National Natural Science Foundation of China (82230045) (Z.W.), (32271066, 82471532) (D.‐D.S), (82501811) (Y.‐D.Z.), Shanghai Science and Technology Committee (23QA1408300, [2023]24) (D.‐D.S), (2025CSJZN01700) (Z.W.), Shanghai Key Laboratory of Mental Disorders Translational Research (No.13dz2260500) (Z.W.), and Shanghai Jiao Tong University School of Medicine (25KCPYYB28) (Y.L.).

## Conflicts of Interest

The authors declare no conflicts of interest.

## Supporting information




**Supporting File**: advs77836‐sup‐0001‐SuppMat.docx.

## Data Availability

The data that support the findings of this stud are available from the corresponding author upon reasonable request.
