# Supplementary material for: A Gut‐Brain Axis Underlying Succinate‐Associated Compulsive‐Like Behavior
Source: Adv Sci (Weinh). 2026 Sep 27:e77836. Online ahead of print. doi: 10.1002/advs.77836 (PMC13616296; doi:10.1002/advs.77836)
Supplement: Supplementary file 1 [file ADVS-9999-e77836-s001.docx]

Supporting Information

**A gut-brain axis underlying succinate-associated compulsive-like behavior**

*Yi Li, Ying-Dan Zhang, Wei Wang, Xin-Yi Wu, Di Li, Xin Gong, Fei Tian, Meng-Yu Dai, Jian Gao, Zi-Feng Zheng, Zi-Yang Bi, Dong-Dong Shi*, and Zhen Wang**

**Supplemental Figures**

**Figure S1. Impact of succinate administration on body weight, water intake, kinematic parameters and postural parameters.**

(A and B) Body weight (A) and change in body weight (B) during the succinate administration period. (*n* = 10 Ctrl, *n* = 10 SA)

(C and D) Water consumed per day (C) and cumulative water consumption (B) during the succinate administration. (*n* = 10 Ctrl, *n* = 10 SA)

(E and F) Speed (E) and movement energy (F) of each body point during the spontaneous behavior analysis. (n = 10 Ctrl, n = 10 SA)

(G-I) Body length (G), body height (H), and body angle (I) during the spontaneous behavior analysis. (*n* = 10 Ctrl, *n* = 10 SA)

(J and K) Frame distance (J) and ratio of time spent in the central area (K) during the spontaneous behavior analysis. (*n* = 10 Ctrl, *n* = 10 SA)

Each dot represents an individual mouse (E-K). Data are represented as mean ± SEM. Statistical significance was determined by unpaired two-tailed *t*-test (G-H), or two-way ANOVA (A-F). **p* < 0.05.

**Figure S2. Impact of succinate on myeline ultrastructure and exploratory behavior-related calcium activity in the dmPFC, and effect of chemogenetic activation on dmPFC neurons.**

(A-D) Representative images (A), thickness (B), g-ratio (C), and scatter plot of g-ratio versus axon diameter (D) of myeline ultrastructure in the dmPFC. (*n* = 22 images from 3 Ctrl mice, *n* = 23 images from 3 SA mice)

(E-H) Heatmap (E), average ΔF/F ratio (F), peak ΔF/F (G), and AUC (H) of dmPFC glutamatergic neuron calcium signals aligned to exploratory behavior onset. (*n* = 24 trials from 4 Ctrl mice, *n* = 25 trials from 5 SA mice)

(I) Left: Representative images of c-fos expression in the dmPFC. Right: Quantification of c-fos-positive cells in the dmPFC of the mCherry group versus the hM3Dq group. (*n* = 4 mCherry, *n* = 4 hM3Dq)

Each dot represents an individual mouse. Data are represented as mean ± SEM. Statistical significance was determined by nested *t*-test (B, C, G, H), or unpaired two-tailed *t*-test (I). **p* < 0.05; ***p* < 0.01; ****p* < 0.001; *****p* < 0.0001.

**Figure S3. Succinate impact transcriptomic profiles in the dmPFC.**

(A) UMAP plot of single-nuclei from all combined samples, colored by annotated cell clusters.

(B) Split UMAP visualization of cell types in Ctrl and SA groups.

(C) Split UMAP visualization of cell types in each sample.

(D) UMAP visualization of the expression of representative well-known cell-type-specific marker genes.

**Figure S4. ^13^C-succinate *in vivo* tracing, microglial SUCNR1 cKO efficiency and its impact on microglia in the dmPFC.**

(A) Schematic of experimental design for isotope succinate detection following oral gavage with ^13^C_2_-labeled succinate.

(B and C) Proportion of ^13^C-succinate among all isotype of succinate in (B) serum and (C) dmPFC (*n* = 4 ^13^C_2_-succinate).

(D and E) Concentration of ^13^C_2_-succinate in (D) serum and (E) dmPFC (*n* = 3 ^13^C_2_-succinate).

(F and G) Validation of *Sucnr1* cKO efficiency in the dmPFC of *Sucnr1*^fl/fl^;*Cx3cr1*-cre^ERT2^ mice (*n* = 3 Ctrl, *n* = 3 cKO) and *Sucnr1*^fl/fl^ mice injected with AAV2/6M-*Cx3cr1*-cre (*n* = 3 Ctrl, *n* = 3 cKO).

(H) Representative images of IBA-1^+^ cells in the dmPFC in the *Sucnr1*^fl/fl^;*Cx3cr1*-cre^ERT2^ mice compared to *Sucnr1*^fl/fl^ mice after succinate administration.

(I) Quantification of IBA-1^+^ microglia in the dmPFC in the *Sucnr1*^fl/fl^;*Cx3cr1*-cre^ERT2^ mice compared to *Sucnr1*^fl/fl^ mice after succinate administration (*n* = 3 Ctrl, *n* = 3 cKO).

Each dot represents an individual mouse (B-G, I). Data are represented as mean ± SEM. Statistical significance was determined by unpaired two-tailed *t*-test (F, G, I). **p* < 0.05.

**Figure S5. Impact of SDV on body weight, water intake, dmPFC activity, and identification of DVC receiving intestinal neural projections.**

(A and B) Body weight (A) and change in body weight (B) during the succinate administration period. (*n* = 10 Sham, *n* = 5 SDV)

(C and D) Water consumed per day (C) and cumulative water consumption (B) during the succinate administration. (*n* = 10 Sham, *n* = 5 SDV)

(E) Left: Representative images of c-fos expression in the dmPFC. Right: Quantification of c-fos-positive cells in the dmPFC of the SA group versus the Ctrl group. (*n* = 3 Sham, *n* = 3 SDV)

(F) Representative image showing HSV-tdTomato expression in the DVC.

Each dot represents an individual mouse (E). Data are represented as mean ± SEM. Statistical significance was determined by unpaired two-tailed *t*-test (E), or two-way ANOVA (A-D). **p* < 0.05.

**Figure S6. Transcriptomic and metabolic profiles of the small intestine following succinate administration in mice.**

(A) PCA of transcriptomic DEGs between the SA and Ctrl groups. (*n* = 4 Ctrl, *n* = 4 SA)

(B, C) Top enriched GO terms for down-regulated (B) and up-regulated (C) DEGs in the SA groups compared to the Ctrl group.

(D) PLSDA of metabolic DAMs in the SA groups compared to the Ctrl group. (*n* = 5 Ctrl, *n* = 5 SA)

(E) Co-enriched KEGG pathway by both DEGs and DAMs.

(F, G) Correlation between intestinal 5-HT concentration and grooming duration (F), grooming bouts (G) after water spray. (*n* = 5 Ctrl, *n* = 4 SA)

(H) Correlation between fecal succinate concentration and intestinal *Htr4* mRNA expression. (*n* = 4 Ctrl, *n* = 4 SA)

**Figure S7. Succinate demonstrates diagnostic potential and correlates with PFC neural activity in OCD patients.**

(A) ROC curve of serum succinate levels distinguishing OCD patients from HC (AUC = 0.7295, 95% CI [0.6058, 0.8532]).

(B) Spearmen’s correlation between zfALFF of Frontal_Sup_R and serum succinate levels in OCD patients.

(C) Spearmen’s correlation between zfALFF of Frontal_ Medial_L and serum succinate levels in OCD patients.

(D) Spearmen’s correlation between zfALFF of Frontal_Medial_R and serum succinate levels in OCD patients.

Each dot represents an individual OCD patient.

**Supplemental Tables**

**Table S1. Relative expression level of microglial homeostatic markers.**

| gene | p_val | avg_log2FC | pct.1 | pct.2 | p_val_adj |
| --- | --- | --- | --- | --- | --- |
| *Hexb* | 3.03E-15 | -0.27626 | 0.954 | 0.954 | 7.28E-11 |
| *Ctss* | 1.21E-14 | -0.28842 | 0.819 | 0.862 | 2.91E-10 |
| *Trem2* | 1.26E-12 | -0.41314 | 0.489 | 0.572 | 3.04E-08 |
| *Siglech* | 3.57E-08 | 0.253263 | 0.635 | 0.541 | 0.00086 |
| *Ctsz* | 6.29E-06 | -0.29696 | 0.395 | 0.445 | 0.151492 |
| *Fcrls* | 0.000146 | -0.29744 | 0.454 | 0.481 | 1 |
| *Olfml3* | 0.00045 | -0.27781 | 0.355 | 0.388 | 1 |
| *Grn* | 0.000646 | -0.36246 | 0.29 | 0.323 | 1 |
| *Tmem119* | 0.001071 | -0.26852 | 0.361 | 0.393 | 1 |

**Table S2. Spearman correlation coefficients between serum succinic acid level and prefrontal cortex subregion fMRI zALFF metrics in OCD patients.**

| brain region | ρ | *p*-value | *p*-value adjusted  (FDR correction) |
| --- | --- | --- | --- |
| Frontal_Sup_L | 0.400092 | 0.080478 | 0.096574 |
| Frontal_Sup_R | 0.410725 | 0.072036 | 0.096574 |
| Frontal_Mid_L | 0.433334 | 0.056306 | 0.096574 |
| Frontal_Mid_R | 0.513706 | 0.020512 | 0.096574 |
| Frontal_Sup_Medial_L | 0.461299 | 0.040632 | 0.096574 |
| Frontal_Sup_Medial_R | 0.34561 | 0.135559 | 0.135559 |

**Table S3. Spearman correlation coefficients between serum succinic acid level and prefrontal cortex subregion fMRI zfALFF metrics in OCD patients.**

| brain region | ρ | *p*-value | *p*-value adjusted  (FDR correction) |
| --- | --- | --- | --- |
| Frontal_Sup_L | 0.408746 | 0.073555 | 0.088265 |
| Frontal_Sup_R | 0.508816 | 0.021958 | 0.043917 |
| Frontal_Mid_L | 0.573691 | 0.008174 | 0.043917 |
| Frontal_Mid_R | 0.534831 | 0.015111 | 0.043917 |
| Frontal_Sup_Medial_L | 0.37761 | 0.100711 | 0.100711 |
| Frontal_Sup_Medial_R | 0.446463 | 0.048459 | 0.072689 |

**Table S4. Spearman correlation coefficients between serum succinic acid level and prefrontal cortex subregion fMRI zReHo metrics in OCD patients.**

| brain region | ρ | *p*-value | *p*-value adjusted  (FDR correction) |
| --- | --- | --- | --- |
| Frontal_Sup_L | 0.453783 | 0.044465 | 0.082124 |
| Frontal_Sup_R | 0.429924 | 0.058494 | 0.082124 |
| Frontal_Mid_L | 0.499683 | 0.024876 | 0.074627 |
| Frontal_Mid_R | 0.520304 | 0.018682 | 0.074627 |
| Frontal_Sup_Medial_L | 0.293672 | 0.208858 | 0.208858 |
| Frontal_Sup_Medial_R | 0.415543 | 0.068437 | 0.082124 |

**Table S5. MRM parameters for the targeted quantification of metabolites**

| analyte | Q1 (m/z) | Q3 (m/z) | RT (min) | DP (V) | CE (V) | Mode |
| --- | --- | --- | --- | --- | --- | --- |
| L-Tryptophan | 205.04 | 146.06 | 4.87 | 78 | 25 | Positive |
| 5-HTP | 221.08 | 204.03 | 3.18 | 105 | 15 | Positive |
| 5-HT | 177.2 | 160.2 | 3.74 | 18.5 | 14.3 | Positive |
| 5-HIAA | 192.1 | 119 | 5.24 | 104 | 37 | Positive |
| succinate | 387.088 | 234.042 | 2.80 | 17.59 | 85 | Negative |

**Table S6. Key resources table.**

| REAGENT or RESOURCE | SOURCE | IDENTIFIER |
| --- | --- | --- |
| Antibodies | | |
| Rabbit anti-c-fos | Abcam | Cat# ab190289 |
| Mouse anti-c-fos | Abcam | Cat# ab208942 |
| Rabbit anti-Iba1 | Wako | Cat# 019-19741 |
| Rabbit anti-mCherry | Abcam | Cat# ab167453 |
| Rabbit anti-GFP | Abcam | Cat# ab290 |
| Alexa Fluor 488 anti-mouse IgG | Cell Signaling Technology | Cat# 4408 |
| Alexa Fluor 555 anti-rabbit IgG | Cell Signaling Technology | Cat# 4413 |
| Bacterial and virus strains | | |
| *Parabacteroides. distasonis* | BeNa Culture Collection | Cat# ATCC 8503 |
| AAV2/9-CaMKIIα-hM3Dq-mCherry | BrainVTA | Cat# PT-0049 |
| AAV2/9-CaMKIIα-mCherry | BrainVTA | Cat# PT-0108 |
| AAV2/1-hSyn-Cre | BrainVTA | Cat# PT-0136 |
| AAV2/9-hSyn-DIO-hChR2-EGFP | BrainVTA | Cat# PT-4358 |
| AAV2/9-hSyn-DIO-EGFP | BrainVTA | Cat# PT-1103 |
| AAV2/9-CaMKIIα-GCaMP6s | BrainVTA | Cat# PT-0110 |
| AAV2/6M-Cx3cr1-Cre | BrainVTA | Cat# PT-0606 |
| Chemicals, peptides, and recombinant proteins | | |
| Sodium succinate | BBI | Cat# A610889 |
| Ampicillin, sodium salt | BBI | Cat# A100339 |
| Metronidazole | BBI | Cat# A600633 |
| Vancomycin hydrochloride | BBI | Cat# A600983 |
| Neomycin trisulfate salt hydrate | BBI | Cat# A610366 |
| Saccharin | BBI | Cat# A430189 |
| Clozapine N-oxide | Selleck | Cat# S6887 |
| Tamoxifen | Sigma-Aldrich | Cat# T5648 |
| Corn oil | absin | Cat# abs42015838 |
| GR113808 | MCE | Cat# HY-103152 |
| DMSO | Sigma-Aldrich | Cat# 41639 |
| Dimethyl malonate | Sigma-Aldrich | Cat# 136441 |
| 4% paraformaldehyde fixative | Biosharp | Cat# BL539A |
| Optimal cutting temperature (OCT) compound | absin | Cat# abs9756 |
| Quick blocking buffer (PBSTx) | Beyotime | Cat# P0226 |
| Triton-X100 | Sangon Biotech | Cat# A600198 |
| Anti-fluorescence quenching mounting medium (containing DAPI) | absin | Cat# abs9235 |
| Disodium succinate-^13^C_2_ | MedChemExpress | Cat# HY-W015410S |
| Critical commercial assays | | |
| Fast Golgi Staining Kit (Neurons and Glial Cells) | Saint-Bio | Cat# BA1808 |
| RNeasy Mini Kit | Qiagen | Cat# 74104 |
| PrimeScript RT Master Mix | TaKaRa | Cat# RR036A |
| TB Green Premix Ex Taq II | TaKaRa | Cat# RR820A |
| Experimental models: Organisms/strains | | |
| C57BL/6J mouse | Vital River,  GemPharmatech,  Cyagen Biosciences | N/A |
| *Sucnr1*^fl/fl^ mouse | Cyagen Biosciences | N/A |
| *Cx3cr1*-cre^ERT2^ mouse | Cyagen Biosciences | N/A |
| *Hoxb8*^-/-^ mouse | Cyagen Biosciences | N/A |
| Softwar and algorithms | | |
| TopScan | CleverSys | N/A |
| BehaviorAtlas Analyzer | BayONE Scientific | N/A |
| BehaviorAtlas Explorer | BayONE Scientific | N/A |
| pClamp | Molecular Devices | N/A |
| InperStudioMultiColorEVAL15 | Inper | N/A |
| InperStudio | Inper | N/A |
| Inper Plot | Inper | N/A |
| Prism 9 | GraphPad Software | N/A |
| ImageJ | National Institutes of Health | N/A |
| OlyVIA | Olympus | N/A |
| R | R Core team | N/A |
| Other | | |
| Multiclamp 700B amplifiy | Molecular Devices | N/A |
| Fiber photometry system | Inper | N/A |
| 465 nm laser generator | Inper | N/A |

**Table S7. Primers used for RT-qPCR**

| Gene | Forward Primer Sequence | Reverse Primer Sequence |
| --- | --- | --- |
| *Sucnr1* | GCCTGACTTTGTTGGGCTTC | AGATCACAACTGCCAGGACC |
| *Htr4* | GTCTTTCAGACGTGCCTTCC | GGCCACCACATTCCACTGTAT |
| *Gapdh* | AAGGTCGGTGTGAACGGATT | TGAACTTGCCGTGGGTAGAG |
